# Reducing gut microbiome-driven adipose tissue inflammation alleviates metabolic syndrome

**DOI:** 10.1186/s40168-023-01637-4

**Published:** 2023-09-21

**Authors:** N. K. Newman, Y. Zhang, J. Padiadpu, C. L. Miranda, A. A. Magana, C. P. Wong, K. A. Hioki, J. W. Pederson, Z. Li, M. Gurung, A. M. Bruce, K. Brown, G. Bobe, T. J. Sharpton, N. Shulzhenko, C. S. Maier, J. F. Stevens, A. F. Gombart, A. Morgun

**Affiliations:** 1 Department of Pharmaceutical Sciences, College of Pharmacy, Oregon State University, Corvallis, OR USA; 2 School of Biological and Population Health Sciences, Nutrition Program, Linus Pauling Institute, Oregon State University, Corvallis, OR USA; 3 Present address: Oregon Health & Science University, Portland, OR USA; 4 Department of Pharmaceutical Sciences, Linus Pauling Institute, Oregon State University, Corvallis, OR USA; 5 Department of Chemistry, Linus Pauling Institute, Oregon State University, Corvallis, OR USA; 6 Present address: UMASS, Amherst, MA USA; 7 Department of Biomedical Sciences, Carlson College of Veterinary Medicine, Oregon State University, Corvallis, OR USA; 8 Present address: Children Nutrition Center, USDA, Little Rock, AR USA; 9 Chemical, Biological & Environmental Engineering, Oregon State University, Corvallis, OR USA; 10 Department of Animal Sciences, Linus Pauling Institute, Oregon State University, Corvallis, OR USA; 11 Department of Microbiology, Department of Statistics, Oregon State University, Corvallis, OR USA; 12 Department of Biochemistry and Biophysics, Linus Pauling Institute, Corvallis, OR USA

## Abstract

**Background:**

The gut microbiota contributes to macrophage-mediated inflammation in adipose tissue with consumption of an obesogenic diet, thus driving the development of metabolic syndrome. There is a need to identify and develop interventions that abrogate this condition. The hops-derived prenylated flavonoid xanthohumol (XN) and its semi-synthetic derivative tetrahydroxanthohumol (TXN) attenuate high-fat diet-induced obesity, hepatosteatosis, and metabolic syndrome in C57Bl/6J mice. This coincides with a decrease in pro-inflammatory gene expression in the gut and adipose tissue, together with alterations in the gut microbiota and bile acid composition.

**Results:**

In this study, we integrated and interrogated multi-omics data from different organs with fecal 16S rRNA sequences and systemic metabolic phenotypic data using a Transkingdom Network Analysis. By incorporating cell type information from single-cell RNA-seq data, we discovered TXN attenuates macrophage inflammatory processes in adipose tissue. TXN treatment also reduced levels of inflammation-inducing microbes, such as *Oscillibacter valericigenes*, that lead to adverse metabolic phenotypes. Furthermore, in vitro validation in macrophage cell lines and in vivo mouse supplementation showed addition of *O. valericigenes* supernatant induced the expression of metabolic macrophage signature genes that are downregulated by TXN in vivo.

**Conclusions:**

Our findings establish an important mechanism by which TXN mitigates adverse phenotypic outcomes of diet-induced obesity and metabolic syndrome. TXN primarily reduces the abundance of pro-inflammatory gut microbes that can otherwise promote macrophage-associated inflammation in white adipose tissue.

Video Abstract

**Supplementary Information:**

The online version contains supplementary material available at 10.1186/s40168-023-01637-4.

## Introduction

Metabolic syndrome (MetS) is associated with abdominal obesity, hypertension, dyslipidemia, and impaired glucose tolerance. It is an increasing global epidemic [1] leading to type 2 diabetes (T2D) and non-alcoholic steatohepatitis (NASH), recently termed metabolic-associated fatty liver disease (MAFLD) [2–4]. Healthy lifestyle changes and regular exercise along with sustained weight management are the only options to prevent or mitigate severity of the associated diseases as there is no FDA-approved treatment; therefore, there is an urgent need to discover additional approaches to prevent or treat MetS [1, 5]. Polyphenols are abundant organic compounds found in plants and used by Ayurvedic practitioners and in traditional medicine for thousands of years to promote various health benefits. The public has great interest in the potential for using these compounds to protect against obesity, metabolic, and cardiovascular diseases [6–8]. It has previously been shown, both by us and other groups, that both the prenylated flavonoid, xanthohumol (XN), isolated from hops (*Humulus lupulus* L.), and its semi-synthetic derivative tetrahydroxanthohumol (TXN) reduce biomarkers associated with MetS in animal models of diet-induced obesity. Although both XN and TXN successfully reduce inflammation [9–11], obesity, hepatosteatosis, and insulin resistance [12–16], TXN attenuates these symptoms more effectively than XN [16]. Previously, we showed TXN treatment decreases expression of many pro-inflammatory cytokines and hypothesized TXN reduces the infiltration of inflammatory macrophages into adipose tissue [17]. Furthermore, previous studies indicated the gut microbiome as a key factor in the beneficial effects of these compounds [17, 18]; however, the causative microbes and the molecular mechanisms by which they affect the host remain unknown. Therefore, we acquired transcriptomic data from the liver, adipose tissue, and ileum, 16S rRNA sequencing of the fecal microbiome, fecal bile acid levels, and systemic metabolic markers from mice fed a low-fat diet (LFD), high fat diet (HFD), or HFD containing XN or TXN from our previous study [16]. We integrated this data using a data-driven systems biology approach Transkingdom Network Analysis. Through reconstruction and interrogation of a transkingdom network, followed by additional in vivo and in vitro experiments, we established TXN mediates its primary therapeutic effect by repressing obesogenic diet-associated microbes (e.g., *Oscillibacter* sp.), which consequently decreases metabolically harmful macrophage-associated inflammation in adipose tissue.

## Results

### TXN more effectively restores metabolic alterations induced by HFD than XN

**Fig. 1:**
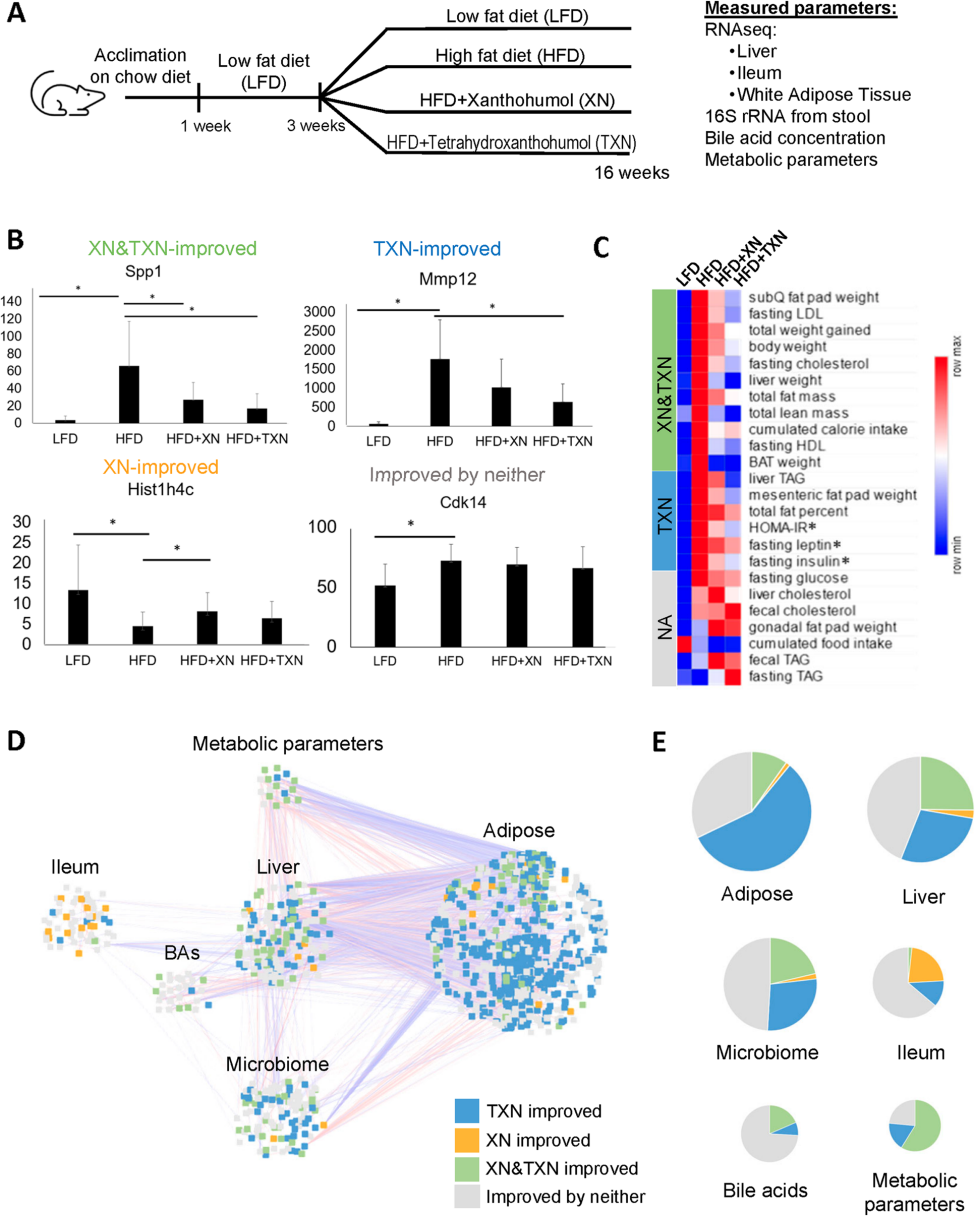
TXN has major effects on gene expression in white adipose tissue.** A** Overview of the mouse experiments, duration, and measurements. The study contained three treatment groups: low fat control diet (LFD), high fat diet (HFD), high fat diet with XN supplementation (HFD+XN), and high fat diet with TXN supplementation (HFD+TXN). Phenotypic parameters, fecal bile acid composition, fecal 16S rRNA gene sequences, and tissue transcriptomes were determined. **B** Each parameter was categorized into one of four different groups (XN-improved, TXN-improved, XN&TXN-improved, improved by neither). Shown here is an example of the expression of one gene from each category, with values in quantile normalized counts per million (CPM). **C** The majority of measured phenotypes (especially those related to glucose homeostasis and adiposity) are reduced by TXN in an opposite way to the direction of change in the HFD group versus LFD (Mann–Whitney *P* < 0.05, * = Mann–Whitney *P* < 0.05 in HFD and < 0.1 in TXN). Colors represent the average of median normalized values of each group, relative to the other groups in the row (e.g., the darkest blue color indicates the lowest mean while dark red indicates the highest). **D** A transkingdom multi-organ network was reconstructed. Nodes in the network represent features that are significantly changed between HFD and LFD. Edges represent Spearman correlations between parameters (red is a positive association, blue is negative). Nodes are colored based on the treatment effect category as described in the Fig. 1B examples. **E** The proportion of parameters in each treatment effect category described in Fig. 1D. Size of the pie chart corresponds to the number of nodes for each data type in the network

To determine the effects of TXN in restoring metabolic alterations induced by a HFD, we used a diet-induced mouse model of obesity and metabolic disease. We randomly assigned 10 mice to one of five groups: LFD, HFD, HFD with added XN (0.035%, low XN), HFD with added XN (0.07%, high XN), and HFD with added TXN (0.035%) for 16 weeks total [16]. We omitted the low XN dose group for downstream analyses in this study as it showed little effect on phenotypic outcomes and lost one mouse in the TXN group (*n* = 9). In each group, we measured changes in the transcriptome of liver, ileum, and epididymal adipose tissue (adipose tissue), microbiome composition in stool, bile acid concentrations in stool, and phenotypic parameters associated with metabolic disease (Fig. 1A). We measured weight, plasma lipid levels, plasma leptin/insulin, and plasma glucose levels as part of these phenotypic characteristics [16].

To elucidate differences between XN and TXN treatments, we identified those parameters changed in the direction of LFD by either XN or TXN treatment when compared to the HFD. For example, a phenotypic parameter (e.g., body weight) increased by HFD as compared to LFD and significantly decreased by one of the treatments was considered as improved by that treatment. We used this to classify all parameters into four primary categories: XN-improved, TXN-improved, XN&TXN-improved, and improved by neither (Fig. 1B).

While both XN and TXN improved 11 phenotypic parameters, including fasting LDL, fasting cholesterol, and brown adipose tissue (BAT) weight, only TXN demonstrated a significant improvement of parameters associated with diabetes, including HOMA-IR, fasting insulin, and MAFLD markers like liver TAGs, mesenteric fat pad weight, and total fat percent. TXN also reduced levels of fasting leptin (Fig. 1C). We also classified changes in transcriptomes, bile acids, and microbiota into these four categories. Overall, these results confirmed our earlier observations [13, 16] that TXN is more efficacious than XN in restoring metabolic alterations caused by an obesogenic diet.

### Modeling metabolic disease via a multi-organ transkingdom network

To model HFD-induced metabolic disease, we reconstructed a multi-organ transkingdom network following our previously established method [19] (Fig. 1D). We have previously used transkingdom networks to identify key regulatory parameters for the progression and treatment of diseases [20, 21]. Following reconstruction of the network, 17 out of 24 phenotypic parameters were represented in the network. Among the tissue transcriptomes, adipose tissue was the most prominently changed by HFD, with 1061 genes represented in the network. Liver and ileum had significantly fewer represented genes (159 and 58, respectively) (Supplementary Table 1). In addition, the network included 27 bile acids and 108 microbial ASVs. Strikingly, TXN alone improved expression of more than 50% of the genes in the adipose tissue network, while both XN and TXN improved approximately 10% of genes (Fig. 1E). XN alone only regulated approximately 1% of genes in the adipose tissue. We observed a similar trend of TXN improving the expression of more genes than XN in liver as well. Compared to XN, TXN predominantly modulated the composition of the microbiome (Fig. 1D and E). Both the systemic metabolic markers and prominent effects of TXN on the adipose tissue at a molecular level indicate the key mechanisms of TXN’s metabolic benefits act through the adipose tissue.

### TXN attenuates inflammatory processes involving macrophages in adipose tissue

**Fig. 2:**
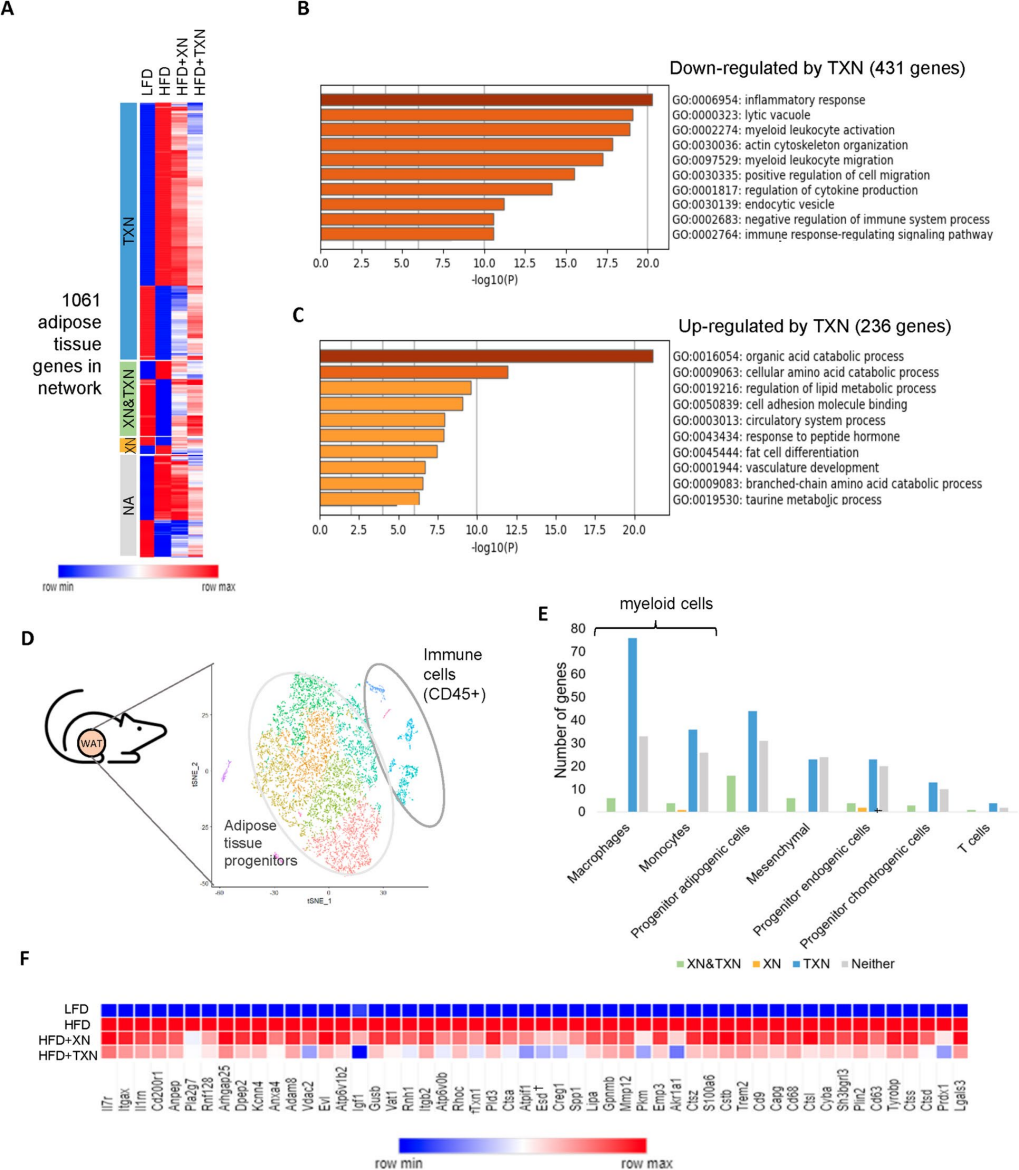
Inflammatory processes in adipose tissue are the primary processes affected by TXN. **A** Heatmap representing expression of each gene in the adipose tissue subnetwork across all treatment groups. Colors represent the quantile normalized log2(CPM+1) mean of each group, relative to the other groups in the row (e.g., the darkest blue color indicates the lowest mean while dark red indicates the highest). **B** Metascape functional enrichment of genes downregulated by TXN showing enrichment for inflammatory processes. **C** Genes up regulated by TXN are enriched for metabolic pathways. **D** The t-SNE plot shows adipose tissue from high fat high sugar diet fed mice (*n* = 5 mice, with 9383 pooled number of cells after preprocessing for quality [Additional file 1]). **E** The number of genes belonging to each cell-type assignment in the adipose tissue subnetwork. TXN improves gene expression primarily in the myeloid cell population. **F** Adipose tissue network genes improved by TXN treatment in the IR-ATM gene signature are shown. Dagger symbol indicates not significant change. Colors represent values calculated in the same way as in **A**

Because 83% of the genes in the network were from adipose tissue and TXN changed the majority (approximately 67%) of adipose tissue genes towards LFD expression levels (Figs. 1E, 2A), we determined which biological functions TXN treatment restored. Genes enriched for inflammatory response, including myeloid leukocyte activation, cytokine production, and phagocytosis were predominantly downregulated by TXN treatment (Fig. 2B). Conversely, TXN upregulated genes enriched for metabolic processes through the restoration of lipid, amino acid, and other metabolic processes (Fig. 2C).

To investigate the reduction in inflammation, we identified those cells primarily responsible for these transcriptomic changes in the adipose tissue. To infer cell type information, we compared our whole tissue transcriptomic data with single-cell RNA sequencing data from adipose tissue of mice fed an obesogenic diet [22]. Adipose tissue genes from the network were classified into specific cell types based on their average expression and fold change in specific cell clusters (Fig. 2D, Additional file 3). TXN primarily affected expression of genes assigned to myeloid cell types (macrophages and monocytes) (Fig. 2E). These genes were enriched for inflammatory processes, including endocytosis and myeloid leukocyte migration (Figure S1A), and regulation of the insulin receptor-signaling pathway.

In addition to the above assignment, to confirm the cell type information, we also took advantage of a previously published dataset (GSE117176) [23] that contains single-cell sequencing of mice on an obesogenic diet (Figure S1B). In accordance with our previous result (Fig. 2D and E), we again found a majority of adipose tissue genes were assigned to metabolic macrophage cells (MC1) (Figure S1C, left panel). Finally, we determined if TXN altered the expression of the genes comprising the transcriptomic meta-signature of macrophages associated with metabolic disease and insulin resistance (named Insulin Resistance associated Adipose Tissue Macrophages: IR-ATMs) (Figure S1C, right panel) [22]. Remarkably, 52 of 62 IR-ATM genes detected in the adipose tissue subnetwork were improved by TXN treatment (Fig. 2F, Figure S1D), supporting a role for TXN in improving the metabolic phenotype of obese mice by decreasing macrophage related inflammation in adipose tissue.

### TXN reduces expression of microbiota-dependent IR-ATM genes

**Fig. 3:**
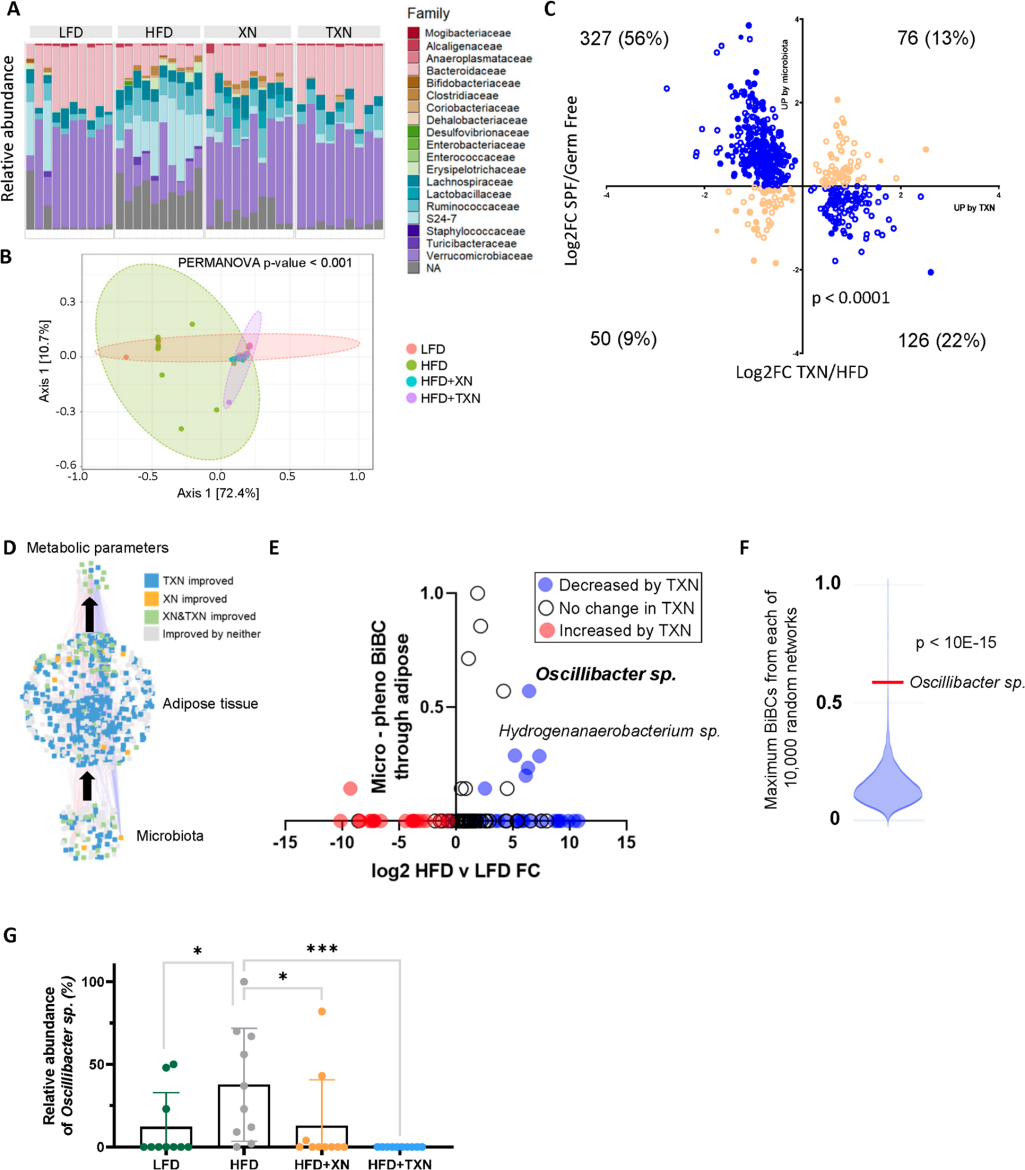
Identification of regulatory microbes causing the inflammatory response. **A** Family level relative abundance plots of microbes across the different treatment groups. **B** PCoA plot of microbiota based on treatment group. **C** The microbiota-dependent up- and down-regulated genes present in adipose tissue and improved by TXN treatment. The x-axis represents log_2_ fold change TXN/HFD and y-axis represents log_2_ fold change SPF/germ-free mice. Blue circles indicate genes whose expression was improved by TXN treatment and microbiota-dependent. Orange circles indicate no reversal in expression direction. The filled circles were assigned to myeloid cells (two-sided Fisher’s exact test, *p*-value < 0.0001). **D** The subnetwork derived from the original network to identify regulatory microbes that affect the host phenotypes by acting through adipose tissue. **E** BiBC between microbes and phenotypes indicates *Oscillibacter* sp. and *Hydrogenanaerobacterium* sp*.* as two regulatory microbes whose levels are both reduced by TXN treatment. **F** An analysis of 10,000 random networks with the same number of nodes and edges as the observed network revealed that the observed BiBC for *Oscillibacter* sp*.* was much higher than due to chance (*p*-value < 10E-15, one-sample Wilcoxon test). **G** The abundance of ASV_61 (identified as *Oscillibacter* sp.) in each treatment group. Brackets indicate the results for specific comparisons. Values are in normalized counts per million. Two-tailed Mann–Whitney *p*-values are shown; *p-value ≤ 0.05; ****p-value ≤ 0.0001

We previously showed that XN requires gut microbiota to mediate its beneficial effects on host metabolism [18]. Therefore, we hypothesized the phenotypic changes seen in TXN-treated mice could result from communication between the gut microbiome and myeloid cells in the adipose tissue. TXN treatment increased the relative abundances of the families *Verrucomicrobiaceae* and *Bacteroidaceae* when compared to the HFD group while decreasing S24-7 (Fig. 3A). Both beta diversity and alpha diversity were also significantly changed (Fig. 3B, Figure S2A).

To identify how TXN acts on host functionally via the microbiota, we used a previous method for discriminating between microbiota-dependent and microbiota-independent effects of antibiotics [24]. For this, we established which TXN transcriptomic alterations in adipose tissue are dependent on microbiota using gene expression data from germ free and SPF mice. We found a large overlap between microbiota-dependent genes and those affected by TXN treatment (Supplementary Table 3). Specifically, out of 667 genes improved by TXN in adipose tissue, 78% were also controlled by microbiota, but in the opposing direction (Fig. 3C). Of note, a majority of these genes were myeloid-specific (Fig. 3C, Supplementary Table 2). As expected, the signature genes for IR-ATMs overlapped with microbiota-dependent genes and 41 of them showed a significant reversal by TXN treatment. Taken together, these findings indicate TXN reduces the effect of HFD-associated microbiota on adipose tissue gene expression.

### *Oscillibacter sp*. is one of the key microbes mediating the beneficial effect of TXN

To identify microbes responsible for the upregulation of the aforementioned IR-ATM signature genes in HFD-fed mice, we restricted our transkingdom network analysis to only the microbiota, adipose tissue transcriptome and metabolic phenotypes. Next, we inferred key microbes with causal contribution to systemic metabolic phenotypes using bipartite betweenness centrality (BiBC). Briefly, BiBC measures the “bottleneck-ness” of a node between two node groups [19]. A high BiBC value indicates a node with regulatory potential that functions as a causal agent in the model under study. In this study, BiBC was calculated between phenotypic parameters and the microbiome through the adipose tissue (Fig. 3D). Microbes of the *Oscillibacter* (ASV_61) and *Hydrogenanaerobacterium* (ASV_70) genera appeared as the top potential causal microbes, as indicated by their decrease with TXN treatment, relatively high BiBC, and high fold-change in HFD (Fig. 3E and G, Figure S2B). Comparing these results with random networks, we found *Oscillibacter* sp*.* is both the top ranked “bottleneck” (BiBC) node and also a highly statistically significant observation (one sample Wilcoxon test *p* < 10^−15^) (Fig. 3E and F). After aligning the 16S rRNA sequence of ASV_61 to NCBI’s RefSeq database using BLAST, we identified *O. ruminantium* and *O. valericigenes* as top hits, respectively. These species are >94% similar, compared to the next most similar taxa (<90%) (Figure S2C). Taken together, these findings indicate TXN’s beneficial effects on adipose tissue and systemic metabolism are mediated by reducing levels of *Oscillibacter* sp. (Fig. 3G).

In a recent study with a diet-induced T2D mouse model, we found that *Oscillibacter* sp. promotes insulin resistance by increasing metabolically damaging macrophages in adipose tissue [22]. There, we demonstrated TLR2 and potentially TLR5 agonists produced by this microbe predominantly mediate the effect of *O. valericigenes* on macrophages. In the current study, we cannot definitively pinpoint which of two species of *Oscillibacter* are present due to limitations of 16S rRNA amplicon sequencing; however, we verified both *O. ruminantium* and *O. valericigenes* are potentially capable of producing Tlr2 and Tlr5 agonists. We identified and aligned the probable *Oscillibacter* species’ protein sequences involved in the biosynthetic processes of TLR2 and TLR5 agonists to known lipoprotein synthesis proteins (*Lgt*, lipoprotein diacylglyceryl transferase, PDB accession code 5AZB) [25] and *LspA*, lipoprotein signal peptidase from *E. coli*) and flagellin synthesis with export apparatus (*FlhAB*, flagellar biosynthetic proteins), respectively (Figure S2D). Thus, reducing the abundance of these microbes in the gut can reduce the level of TLR agonists.

As described above, we inferred that TXN decreases the IR-ATM signature by reducing *Oscillibacter* sp. Furthermore, in our recent study [22], we showed that IR-ATMs negatively affect systemic metabolism via damage of mitochondria in adipose tissue. Thus, we hypothesized TXN may restore adipose tissue mitochondrial function in mediating its beneficial downstream effects. To support our hypothesis, we determined the effect of TXN on mitochondrial gene expression in the adipose tissue. First, we found that genes improved by TXN in the adipose tissue were enriched for mitochondrial processes such as antioxidant activity and oxidoreductase activity (Figure S3A).

Next, we identified 87 mitochondrial genes (MitoCarta 3.0 [26]) in the adipose tissue network that were increased by TXN. Among the genes predicted to mediate the effect of myeloid cells on systemic metabolism (high BiBC between myeloid cells and phenotypes), we observed a trend of enrichment for mitochondrial function. For example, among these predicted regulatory genes, there were several genes with well-known mitochondrial function such as thioredoxin reductase [27], glutathione transferase [28], and many coenzyme A-related genes [29] (Figure S3B). Taken together, our previous findings and these results indicate TXN treatment may improve mitochondrial function in the adipose tissue, similar to what we previously reported for the liver [13].

### Macrophage genes induced by *Oscillibacter* are inhibited by TXN

**Fig. 4:**
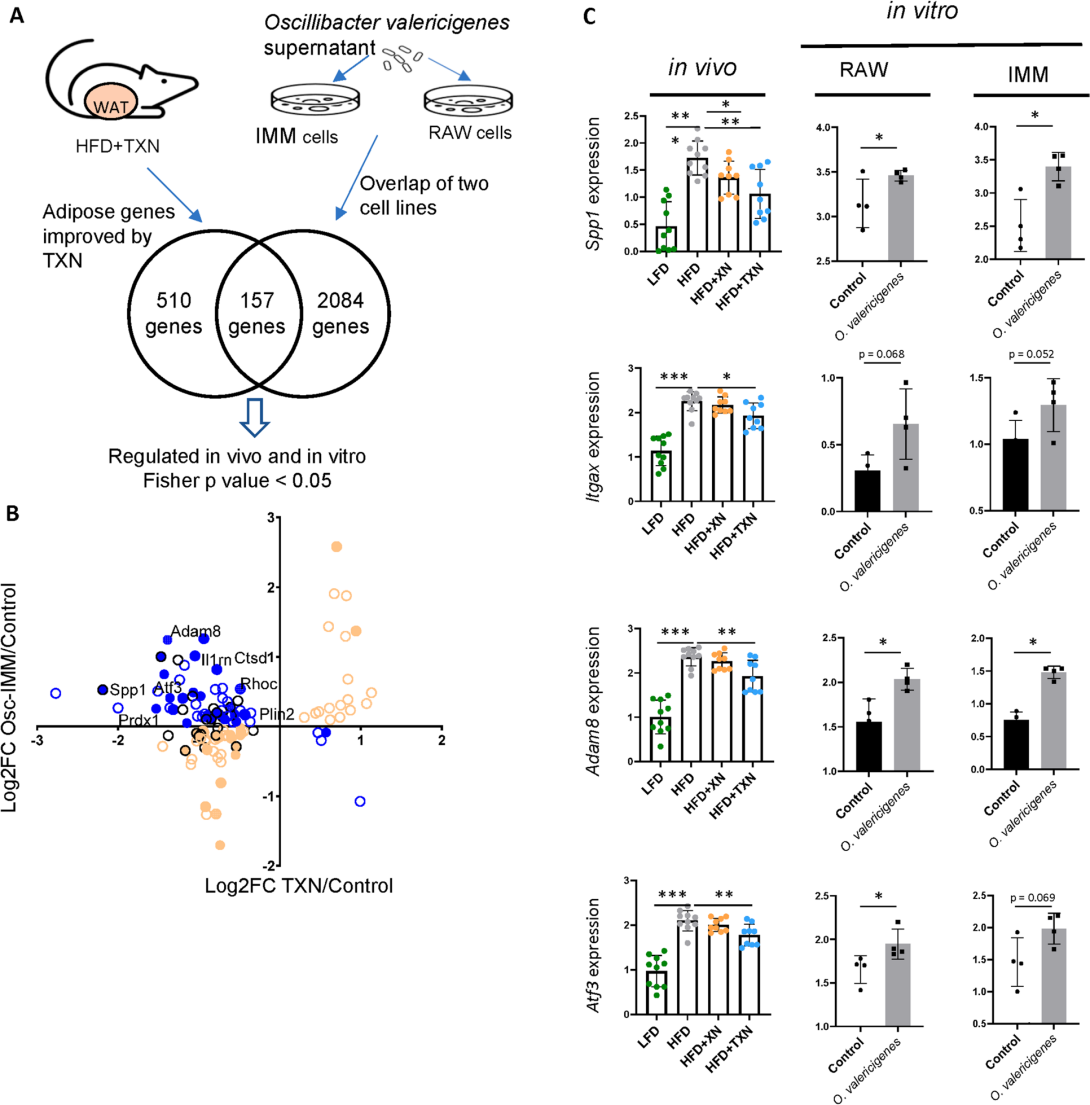
*O. valericigenes* treatment of macrophage cell line increases the expression of IR-ATM genes whose expression was improved by TXN in vivo. **A** Treatment of macrophage cell lines IMM and RAW 264.7 with *O. valericigenes* regulated expression of 2241 genes (one-sided Fisher’s exact test; FDR-adjusted *p*-value < 0.05), of which 157 were reversed by TXN treatment in the adipose tissue of mice fed HFD (HFD+TXN). **B** The XY-plot shows genes significantly improved by TXN treatment in vivo with concordant regulation in both macrophage cell lines (individual comparison *p*-value < 0.1). Blue circles indicate those genes that were improved by TXN treatment and were microbiota-dependent. Orange circles indicate no change in expression direction. The filled circles indicate those genes assigned to myeloid cells. The black circles indicate IR-ATM signature genes (one-sided Fisher’s test, *p*-value < 0.05). **C** Expression of representative genes decreased by TXN treatment in the adipose tissue in vivo and increased with *O. valericigenes* treatment in vitro. Values are normalized counts per million. *Mann–Whitney *p*-value < 0.05; **p-value < 0.01; ***p-value < 0.001

To assess the inflammatory effects of *O. valericigenes*, we exposed two mouse macrophage cell lines, RAW 264.7 and IMM (immortalized mouse macrophage cells) to cell-free supernatant from this microbe **(**Fig. 4A). *O. valericigenes* supernatant altered the transcriptome of 2241 genes in both cell lines. Of those, TXN regulated expression of 325 genes in RAW 264.7 cells that belonged to the adipose tissue network and were microbiota-dependent. In IMM cells, 268 genes fit the same criteria (Figure S4A). Overall, of the 667 adipose tissue genes improved by TXN, 157 overlapped with the genes regulated by *O. valericigenes* supernatant in both IMM and RAW264.7 cells (Fig. 4A). Specifically, supernatant exposure induced IR-ATM signature genes including *Itgax*, *Adam8*, *Spp1*, *Plin2*, and *Prdx1* as well as *Atf3* (Fig. 4C, Supplementary Table 3), a transcription factor that controls critical effector molecules of these macrophages [22]. Of note, 79 of these 157 genes, including many IR-ATM signature genes, were significantly increased in both *O. valericigenes* supernatant-exposed macrophage cell lines and were reduced by TXN treatment in vivo in the adipose tissue myeloid cells (Fig. 4B, figure S4B). Additional experiments demonstrated the expected loss of induction of proinflammatory molecules in vitro when diluting *O. valericigenes* supernatant (Figure S5A).

**Fig. 5:**
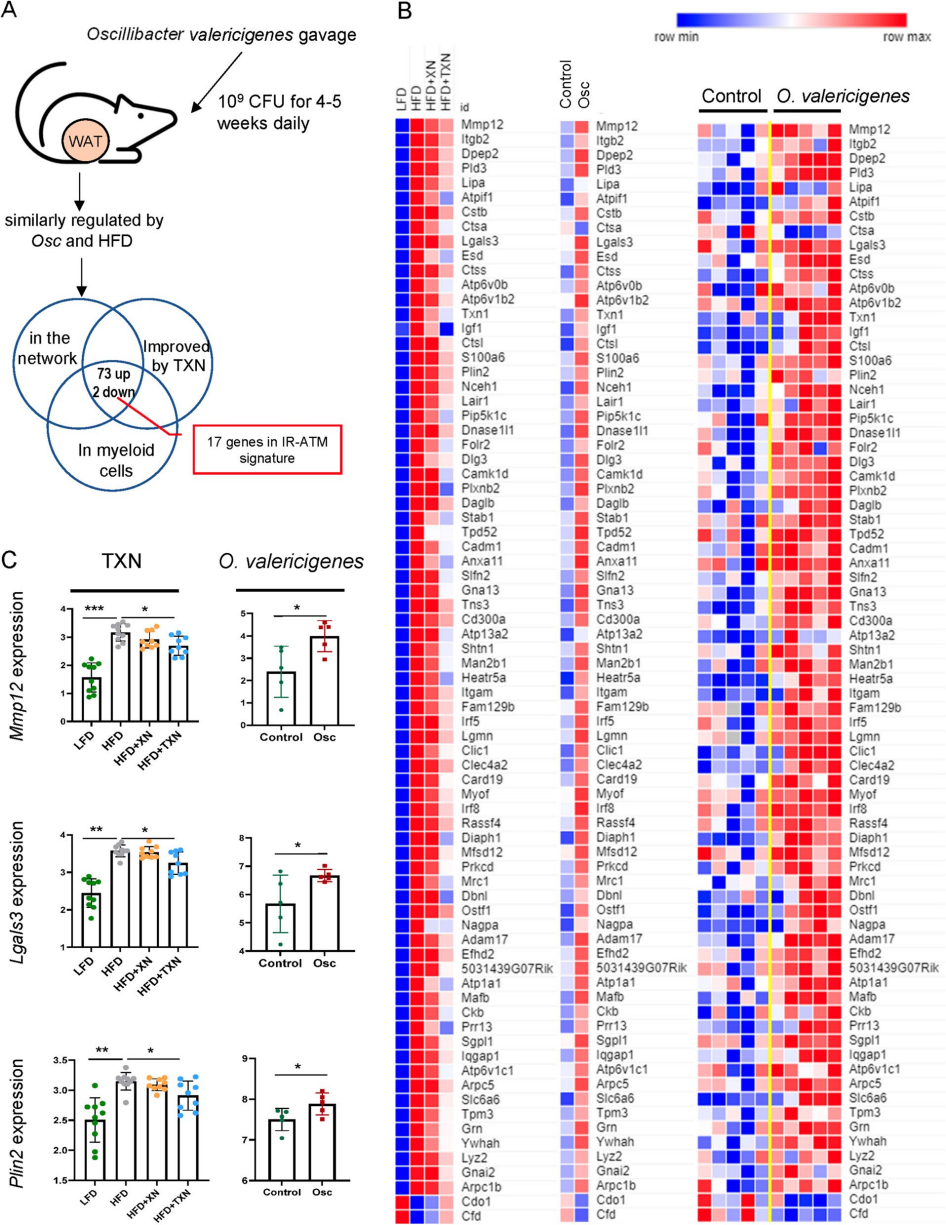
*O. valericigenes* treatment increases the expression of IR-ATM genes in mouse white adipose tissue that are improved by TXN treatment. **A** Outline of *O. valericigenes* experiment and subsequent gene expression analysis from WAT (*n* = 5 per group). The Venn diagram shows 75 genes from myeloid cells overlapping between similar effects of HFD and *O. valericigenes* with opposite effects of TXN. WAT genes from the network in Fig. 1D. **B** Expression of adipose tissue genes increased by HFD and decreased by TXN treatment in mice (left panel) vs the same adipose tissue genes following *O. valericigenes* supplementation of mice (right panel). The middle panel shows the mean of the samples in the right panel. Colors represent the quantile normalized log2(CPM+1) mean of each group, relative to the other groups in the row (e.g., the darkest blue color indicates the lowest mean while dark red indicates the highest). **C** Comparison of in vivo adipose tissue gene expression in two sets of experiments. One experiment used varying diets (low fat diet and high fat diet) and interventions (XN and TXN treatment) while the other used only a normal chow diet with or without *O. valericigenes* supplementation. Shown are examples of adipose tissue genes (in the IR-ATM signature) decreased by TXN treatment but induced by O. valericigenes (Osc) supplementation in vivo. Values are in normalized counts per million. *Mann–Whitney *p*-value < 0.05; **p-value < 0.01; ***p-value < 0.001

To support our in vitro findings, we compared expression of adipose tissue genes from mice treated with TXN versus those from mice fed a control diet with mice supplemented daily with *O. valericigenes* (10^9^ CFU) for 4–5 weeks in our previous study [22]. We found that 75 genes (73 up- and 2 down-regulated by the microbe) out of 98 genes modulated by *O. valericigenes* administration were improved by TXN treatment in vivo. Of these genes, 17 (e.g., *Mmp12*, *Lgals3*, and *Plin2*) belonged to the IR-ATM signature (Fig. 5A–C, Supplementary Table 4). Of note, another microbe (*L. gasseri*) common to microbiota of HFD-fed mice but not predicted by our analysis as potential inducer of adipose inflammation (see “Methods”) did not stimulate increase of expression of immune genes either in vitro (Figure S5A, lower panel, Supplementary Table 5) or in vivo (Figure S5B).

**Fig. 6:**
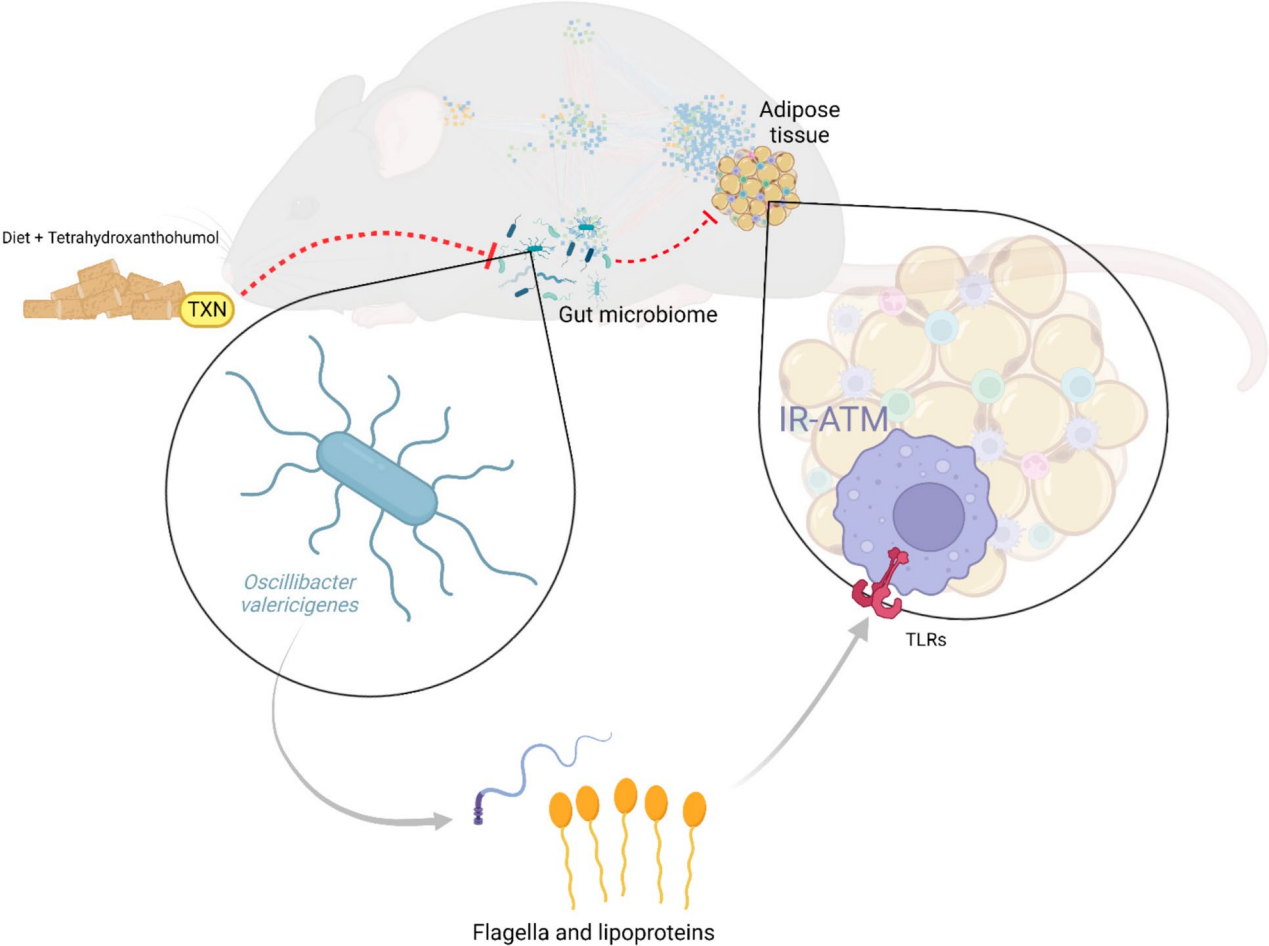
Summary figure. Consumption of TXN affects the composition of the gut microbial community, reducing *Oscillibacter* spp. (known to produce TLR agonists such as flagellin and lipoproteins), which in turn leads to a reduction of metabolically damaging insulin resistance associated adipose tissue macrophages (IR-ATMs)

Taken together, our results indicate the interaction between the gut microbiota and adipose tissue comprises the key cellular and molecular events mediating beneficial effects of TXN on systemic metabolism. Specifically, we posit that reduction of *Oscillibacter* (and potentially other bacterial producers of Tlr2/5 ligands) levels and the consequent decrease and/or reprogramming of metabolically harmful IR-ATMs represents one of the main mechanisms of TXN therapeutic action in MetS (Fig. 6).

## Discussion

Xanthohumol (XN) and its derivatives mitigate diet-induced obesity-related characteristics of MetS in mice by improving impaired glucose and lipid metabolism [13, 30–32]. XN also exhibits hepatoprotective effects in animal models of MAFLD and other liver-damaging conditions [33, 34]. Accumulating studies indicate these prenylated flavonoids may mediate their beneficial effects via polypharmacological mechanisms of action, which could increase their effectiveness in treating disease. Previous studies link the anti-obesity and MetS benefits of XN and its derivatives to possibly increasing energy expenditure through mild mitochondrial uncoupling and increasing locomotor activity [13, 35, 36]. XN improves diet-induced obesity and hepatosteatosis by inhibiting expression of and inactivating maturation of sterol regulatory element-binding proteins, thus reducing the expression of genes involved in fatty acid and cholesterol biosynthesis [14, 37]. Evidence also suggests XN and even more so, TXN, attenuates hepatosteatosis by acting as antagonists of PPARγ [16]. Improvement in glucose homeostasis by XN was attributed to AMPK activation in the mouse liver [13, 38]; however, we did not observe this with TXN [13]. We also found improvements in glucose metabolism by XN required the microbiota [18]. Furthermore, administration of XN and TXN altered the gut microbiota composition and bile acid metabolism in mice implicating these changes in mediating the benefits of XN and TXN [17]. In particular, administering TXN was very effective in changing gut microbiota composition, bile acid metabolism, and reducing adipose tissue inflammation [17]. Despite these numerous studies, there is no consensus which organs and molecular mechanisms contribute the most to the beneficial effects of XN and its derivative TXN.

In this study, to delineate the beneficial effects of TXN, we used a multi-omics transkingdom network analysis of data obtained from diet-induced obese murine models and validated the outcome of the analysis with additional in vitro and in vivo data. TXN acted predominantly on adipose tissue with approximately two-thirds of DEGs altered by TXN treatment. Of those genes whose expression was decreased by TXN, there was significant enrichment for inflammatory processes, implicating myeloid cells, especially macrophages, as an important factor in the disease in part dependent on microbiota.

The connection between gut microbes and obesity-related inflammation leading to MetS remains a poorly characterized phenomenon [39]. In a recent publication [22], we described expansion of *O. valericigenes* among a few other gut microbes in mice fed a high fat, high sugar diet, which increased Mmp12-positive macrophages with a particular transcriptomic signature indicative of a unique cell population of IR-ATMs. In this study, using an HFD in mice in a different facility, our unbiased investigation identified inflammatory macrophages with a very similar signature in the adipose tissue.

Causal inference via our transkingdom network analysis revealed *Oscillibacter* spp. as a key microbe mediating the effects of TXN in decreasing inflammation in adipose tissue and improving symptoms of MetS. From our analysis, we inferred several microbes, including *O. valericigenes* that could activate TLRs and experimentally demonstrated this activation increased expression of numerous IR-ATM genes in vitro in RAW 264.7 and IMM cell lines and in vivo in adipose tissue of mice fed control diet. This activation is consistent with the molecular machinery of this pathobiont. We found *Oscillibacter* contained genes encoding proteins with high sequence identity to lipoprotein synthesis pathway proteins responsible for synthesizing lipoprotein, a TLR2 agonist [40], along with flagellin synthesis and assembly machinery proteins responsible for synthesizing flagellin, a TLR5 agonist [41]. TXN-treatment decreased expression of these proinflammatory genes, characteristic of IR-ATMs in adipose tissue of mice fed an HFD. Thus, these experiments support TXN’s mechanism of action in the adipose tissue which is predominantly related to dampening macrophage-mediated inflammation. Spp1, Cd9, Trem2 and Mmp12, [22, 42–45] and other well-known IR-ATM genes were among the ones increased by the microbial supernatant while TXN significantly decreased their expression in adipose tissue. Taken together, these findings reveal that TXN benefits the host by acting on IR-ATMs via changes in the composition of the gut microbiota. Recent studies have focused on various aspects of these specific macrophages (IR-ATMs), which were also referred as lipid-associated macrophages (LAMs) [43], vasculature-associated macrophages (VAMs), double-positive macrophages (DPs) [45], CD9+ macrophage cells [42], or metabolic macrophages [46]. Each of these studies related this group of macrophages to insulin resistance and inflammation. Our findings may have translational potential as we previously showed this IR-ATM signature shows a strong overlap with that found in a meta-analysis of T2D patients [22]; suggesting TXN treatment could benefit obese patients with Type 2 Diabetes.

Current literature provides compelling evidence for a model of MetS pathogenesis that involves more than a few microbes acting on different mammalian pathways and organs [47–49]. Accordingly, while the sample size of this study allowed us to infer only one top bacterium (*Oscillibacter*) clearly contributing to the beneficial effect of TXN, we also observed a similar functional trend for a few other microbes decreased by TXN (Fig. 3E). Some (e.g., *Hydrogenanaerobacterium*) were previously reported by us [22] and others [50] to be associated with MetS. Moreover, a previous study demonstrated that at least some of the positive outcomes of XN are accomplished by increasing *Akkermansia muciniphila*, which we also found increased by TXN [18]. However, we did not observe any significant effect of TXN treatment on other known beneficial microbes, such as Lactobacillus or Lactococcus [20]. These results suggest that when microbiota mediate the effect of a given treatment, the specific cellular/molecular mechanism would strongly depend on the presence/absence of causally contributing microbes in the underlying microbial community.

There are some limitations with respect to the design of this study. First, we are unable to test the effect of TXN in germ-free (GF) mice, since GF mice do not develop inflammation as a result of a lack of microbiota [51–53]. Additionally, this study relies on the assumption that transcriptomic changes are reflective of the abundance of functional proteins, so proteomic data was not investigated.

In conclusion, XN and its derivatives mediate their beneficial effects against obesity and MetS through several mechanisms of action. However, to the best of our knowledge, this is the first study identifying a predominant mechanism of action for TXN in reducing microbial community members (e.g., *Oscillibacter* sp.) which promote metabolically damaging adipose tissue macrophages in response to an obeso-/diabeto-genic diet. Though we identified a new mechanism explaining benefits of TXN on adipose tissue, one should note that TXN also affects intestine, liver, bile acids, and additional microbiota, warranting further investigation in future studies.

## Methods

### Animals and diets

Animals and diets were described previously in detail [16]. Briefly, after acclimation, 60 8-week-old C57BL/6J mice purchased from The Jackson Laboratory (Bar Harbor, ME, USA) were randomly assigned to one of five diet groups (*n* = 12/group): (1) low fat diet (LFD), (2) high fat diet (HFD), (3) HFD + 0.035% XN (LXN), (4) HFD + 0.07% XN (HXN), or (5) HFD + 0.035% TXN (TXN) for 16 weeks. Body weight and food intake were assessed weekly [16]. Upon conclusion of the study, mice were fasted for 6 h during the dark cycle, euthanized, and blood and tissues collected as described previously [16]. One mouse from the TXN group died and was excluded from subsequent analysis. For isolation of stromal cells from visceral adipose tissue, 7-weeks old, specific pathogen-free (SPF), C57BL/6J male mice were purchased from The Jackson Laboratory. After 1 week of acclimation, mice were switched to either a western diet (WD) D12451 containing 45% lard and 20% sucrose or a matched normal diet D12450K (ND) produced by Research Diets (New Brunswick, NJ USA). Mice were maintained on these diets for 8 weeks. All animals were housed in the Laboratory Animal Resources Center (LARC) at Oregon State University in a controlled environment (12-h daylight cycle) with ad libitum access to food and water. All animal experiments were approved by the Institutional Animal Care and Use Committee (IACUC) at Oregon State University and performed in accordance with the relevant guidelines and regulations as outlined in the Guide for the Care and Use of Laboratory Animals.

### Administration of *Oscillibacter valericigenes* to mice

Experiments were done as described in our previous study [22]. WAT gene expression data generated there was used to find overlap with the TXN-regulated WAT genes in this study.

### Measurement of plasma and metabolic parameters

Measurements of fasting LDL, cholesterol, glucose, insulin, and leptin, glucose tolerance, liver TAGs, and weights of brown adipose tissue (BAT) and mesenteric fat pads were performed as described previously [16, 31].

### Quantification of fecal bile acids

Total fecal samples were collected for a 3-day period to calculate an average fecal output for that period. Fecal material was lyophilized and ground into powder for extraction. Powdered feces (50 mg) were mixed with a 10 ml volume of bile acid internal standards (CA-d4, 10 mg/mL; DCA-d4, 10 mg/mL; GCDCA-d4, 2 mg/mL; CDCA-d4, 2 mg/mL; TCDCA-d4, 2 mg/mL). Methanol (1.5 mL) was added and samples were shaken in a Precellys homogenizer for 1 h (Bertin Instruments, Rockville, MD, USA) containing ceramic beads, centrifuged at 16,000 rpm in a microfuge for 10 min and supernatant collected. Samples were extracted two more times as described above. Supernatants were pooled, evaporated under a vacuum, and reconstituted in 0.5 mL 50% methanol and randomized for data acquisition. UPLC was performed using a 1.7 μm particle, 2.1 mm × 100, CSH C18 column (Waters, Milford, MA, USA) coupled to a hybrid triple quadrupole linear ion trap mass spectrometer (AB SCIEX, 4000 QTRAP). LC and MS conditions are described previously [54]. BAs were identified by matching their retention time, isotopic pattern, exact mass of the [M-H]- ion, and fragmentation pattern with a panel of authentic standards (IROA Technologies, Sea Girt, NJ, USA).

### Tissue RNA extraction and library preparation

Fresh dissected tissues were flash frozen in liquid nitrogen and stored at −80°C. Libraries for Illumina sequencing were prepared as described previously [16]. Approximately 6.6 million reads were obtained per liver sample. Reads for adipose tissue and ileum, sample sequence alignment and gene counts, and identification of differentially expressed genes were performed as described previously [16].

### RNA-Seq sequence alignment and gene counts

Sequences were processed to remove the adapter, polyA, and low-quality bases using the fastx_clipper command in the FASTX-Toolkit v0.0.13 (FASTX-Toolkit: FASTQ/a short-reads pre-processing tools. http://hannonlab.cshl.edu/fastx_toolkit/), followed by BBDuk (bbduk.sh) parameters of *k* = 13, ktrim = r, forcetrimleft = 11, useshortkmers = t, mink = 5, qtrim = r, trimq = 10, minlength = 20. Reads were aligned to mouse genome and transcriptome (GENCODE GRCm38) using STAR (v020201) with default parameters. Number of counts per million (CPM) for mouse genes were quantified using HTSeq (v 0.6.0) and quantile normalized. The resulting values were then log2-transformed prior to conducting statistical tests. To avoid log2 values of 0, a pseudocount of 1 was added to each normalized CPM before log2 transformation. Differentially expressed genes were found using BRB-ArrayTools (https://brb.nci.nih.gov/BRBArrayTools/). Following sequencing, two samples from the LFD, HFD, and TXN groups, along with three samples from the XN group, were removed due to sequencing quality issues. Additionally, one mouse in the TXN group died during the study and thus was not sequenced.

### DNA extraction and 16S rRNA gene libraries preparation

Fresh fecal pellets were collected from each mouse, frozen in liquid nitrogen, and stored at −80°C at the end of the feeding study. DNA was extracted as described previously [17, 18]. Sequencing of the 16S rRNA genes was performed by the Genomics and Cell Characterization Core Facility at the University of Oregon. Briefly, custom-designed PCR primers that contain dual-indexed adapters were used to amplify the V3–V4 (806R/319F) region using NEBNext® Q5® Hot Start HiFi PCR Master Mix (New England Biolabs, Beverly, MA, USA). Samples were purified by two repeated 0.8X-ratio magnetic bead clean ups to remove all traces of primer. The optimal number of cycles were determined by initial testing on a subset of samples. A no-template control and a ZymoBIOMICS D6305 microbial community DNA standard were included (Zymo Research, Irvine, CA USA). Paired-end 300 bp sequencing was performed on an Illumina MiSeq PE300 (v3 mode) with PhiX added in to about 25%. Approximately 50–60K reads per sample were achieved. Identification of gut microbial amplicon sequence variants (ASVs) followed by chimera removal was performed with DADA2 (v1.16) [55]. Taxonomy assignment was performed using the Ribosomal Database Project’s Training Set 16 and the 10.28 release of the RDP database [56]**.** Raw sequencing data was deposited in the NCBI Gene Expression Omnibus.

### Identification of Lactobacillus *gasseri*

A candidate commensal that did not exhibit increased abundances in HFD, nor was affected by TXN, and that was not predicted by our methods to be important for a pro-inflammatory response was identified (ASV_8). ASV_8 was annotated by SILVA as *Lactobacillus* sp. BLAST was further used to annotate the ASV as *L. gasseri* ATCC33323, with a 99.74% identity (E-value = 0.0) to the representative 16S amplicon sequence.

### Bacterial cultures and cell-free supernatant (CFS) preparation

*O. valericigenes* DSM 18026 was cultured in peptone yeast glucose (PYG) medium (AS 825; Anaerobe Systems) for 4 days at 30°C in an anaerobic GasPak jar (BD), then harvested, centrifuged at 4000 rpm for 20 min at 4°C, supernatant was filtered through 0.2 μM low protein binding filter, aliquoted in 2 ml tubes, then stored at −80°C. *Lactobacillus gasseri* ATCC33323 was grown in MRS broth in an anaerobic GasPak jar (BD) for 24 h at 37°C and CFS was prepared as described above.

### Macrophage cell culture

Cell culture of THP-1 and IMM cells was performed as described previously [22]. Immortalized Mouse Macrophages (IMM) were originally generated and shared with us by Dominic de Nardo [57] and grown in DMEM media (4.5 g/liter glucose) supplemented with 10% FBS, 1% penicillin/streptomycin, and 20 mM HEPES. A total of 50,000 cells/well were seeded in 5 wells per treatment in 96-well plates overnight in complete DMEM medium with serum and incubated at 37°C with 5% CO2. Cells were then washed with DPBS and stimulated in DMEM media (without serum) for 6 h with *O. valericigenes* CFS (or PYG media as control) or *L. gasseri* ATCC33323 CFS (or MRS media as control). Cells were then collected in RLT lysis buffer (Qiagen) by scraping before being stored at −80°C until RNA extraction. Titrations were performed from an original 5% v/v dilution of *O. valericigenes* or *L. gasseri* CFS in DMEM (serum free), serial 5-fold dilutions were prepared, and 200 uL of media was added to each well. Experiments were repeated 3 times.

### qPCR gene expression and analysis

Complementary DNA (cDNA) was prepared from 1 ug of extracted RNA using the qScript XLT cDNA synthesis reverse transcription kit (QuantaBio). qPCR was performed in triplicate wells for each gene using the AzuraQuant Green Fast qPCR HiRox master mix (Azura genomics: AZ-2050) and the StepOnePlus Real Time PCR system/software (Applied Biosystems). Raw cycle threshold (CT) values of genes of interest from qPCR runs were normalized to CT values of the housekeeping gene (Polr2c) via delta CT method before calculation of relative expression using the 2−ΔCT method. Relative expression levels of genes were median-normalized and fold changes (over unstimulated controls) were log2 transformed. To compare means of gene expression in CFS treated cells versus the respective control, one-sided unpaired parametric *t*-tests were performed in GraphPad Prism 9 (version 9.5.1).

Primer table

**Table:** 

Gene	Forward (5’→3’)	Reverse (5’→3’)
Polr2c (housekeeping gene)	CTCACCGAAGAGAACGTCAAG	TCGATGGCTATTATGGGCACC
Mmp12	CAGGTCACACACACATAGTTACACA	AAACCAGTTGGCCTCTGAAC
Atf3	GAGGATTTTGCTAACCTGACACC	TTGACGGTAACTGACTCCAGC
Spp1	CAATGAAAGCCATGACCACA	TCCGAGTCCACAGAATCCTC

### Transkingdom multi-organ network construction and interrogation

The transkingdom network was reconstructed generally following previously described guidelines [19]. More specifically, the Mann–Whitney *p*-value and Fisher’s combined *p*-value were calculated for each measured parameter (between HFD and LFD, XN and HFD, and TXN and HFD). FDR was then calculated separately for each data type and for each tissue. *p*-value and FDR thresholds were then applied.

Correlations were performed within each data set (LFD, HFD, HFD+XN, and HFD+TXN). Spearman correlations were performed on the remaining parameters, followed by combining the resulting *p*-values using Fisher’s z transformation of correlations in the R package meta v5.5.0 [58]. Correlations were then filtered for a maximum *p*-value of 0.5 and combined *p*-value of 0.05, except in bile acids-adipose tissue, bile acids-liver, and microbiota-ileum, where a combined *p*-value threshold of 0.1 was used. Correlations and subsequent FDR calculations within and between each pair of data types (micro-micro, micro-gene, gene-phenotype, gene-gene, etc.) were performed separately and then integrated into a final network. FDR was further reduced following the removal of unexpected correlations, which are caused by the violation of correlation inequalities [59]. Within-omic correlations had more stringent statistical thresholds than between-omic correlations.

We also accounted for two other network parameters. First, network sparsity which we compared the edge:node ratio of the reconstructed network to the edge:node ratio of a complete network with the same number of nodes, using the formula below.

$$\mathrm{Deviation}=\frac{\left|\mathrm{Observed edge}:\mathrm{node ratio}-\mathrm{Expected edge}:\mathrm{node ratio in complete graph}\right|}{\mathrm{Expected edge}:\mathrm{node ratio in complete graph}}$$

Second, to reduce the bias of positive correlations [60], we chose our statistical thresholds (below arbitrary chosen FDR, see above) that the observed negative to positive signs of correlation ratio in a network would be the closest to a complete graph with no noise that fulfills correlation inequalities criteria [59].

To establish the most critical microbes for the transition to a diseased state following prolonged consumption of a high fat diet, we employed the use of bipartite betweenness centrality (BiBC). As described previously [19], BiBC is a measure of how vital a node is for information to get from one group in a network to a second group. It identifies the nodes that are the bottlenecks between groups, allowing for inference of these nodes as probably targets for treatment. This inference was then experimentally validated [20, 22]. In this study, we used the microbiota and host phenotypes as our two node groups, allowing us to identify which microbiota are the top mediators of this disease.

### Random network analysis

Random networks were created and analyzed as described previously [61]. In brief, 10,000 Erdos-Renyi random networks were created, using the same nodes in the real microbiota-adipose tissue-phenotype networks and the same number of edges (7271). BiBC was calculated between microbiota and phenotypes for each random network and the maximum node BiBC was extracted. These were then scaled on a 0 to 1 scale and compared to the actual calculated BiBC of *Oscillibacter* sp. in the reconstructed network.

### Isolation of stromal cells from visceral adipose tissue

Epididymal adipose tissue from four C57BL/6J male mice were isolated separately, mechanically dissected, then minced in Hanks’ Balanced Salt Solution (HBSS; ThermoFisher Scientific, Waltham, MA USA) containing calcium, magnesium and 0.5% BSA and 3 mg/ml collagenase type 1 (ROCKLAND, Philadelphia, PA USA). After incubation at 37°C for 1 h, the cell suspension was filtered through 100 $\upmu$ M strainer and then washed with 20 mL DPBS containing 1 mM EDTA and 0.5% BSA (wash buffer). After centrifugation at 700 g for 15 min, the supernatant was removed, and the cell pellet was re-suspended in 5 mL of RBC lysis buffer (TONBO^TM^ A Cytek® Brand, San Diego, CA, USA) and incubated for 10 min at room temperature. Wash buffer (10 mL) was added, and the cell suspension was centrifuged again. The cell pellet was re-suspended in 1 mL FBS containing 10% DMSO and stored at liquid nitrogen until use.

### Single-cell RNA seq from adipose tissue stromal cells

Stromal cells from epididymal fat tissue of four C57BL/6J male mice were isolated as described previously [22] and pooled prior to scRNA-seq. All cells were resuspended in DPBS with 0.04% BSA, and immediately processed for scRNA-seq as follows. Approximately 10,000 cells were loaded for capture onto the Chromium System using the V2 single-cell reagent kit according to the manufacturer’s protocol (10× Genomics, Pleasanton, CA, USA). Following capture and lysis, cDNA was synthesized and amplified as per the manufacturer’s protocol (10x Genomics). The amplified cDNA from each channel of the Chromium System was used to construct an Illumina sequencing library and was sequenced on a HiSeq 4000 sequencing. Illumina base call files (*.bcl) were converted to FASTQs using CellRanger v1.3, which uses bcl2fastq v2.17.1.14. Mouse reference genome mm10 was used to align FASTQ files and transcriptome using default parameters with the CellRanger v1.3 software pipeline as previously reported; this demultiplexes the samples and generates a gene versus cell expression matrix based on the barcodes and assigns UMIs (unique molecular identifier) that enables determination of each of the individual cell from the pooled adipose tissue stromal cell samples which the RNA molecule originated.

### Single-cell RNA seq data analysis

The raw gene expression matrix (UMI counts per gene per cell) was filtered, normalized, and clustered using a standard Seurat version 3.1.0 in in R (https://www.R-project.org/) [62]. Cell and gene filtering were performed as follows: cells with a very small library size (<2500) and a very high (>0.5) mitochondrial genome transcript ratio were removed during quality check. Genes detected (UMI count > 0) in less than three cells were removed. Log normalization and clustering is performed using standard Seurat package procedures. Principal component analysis was used to reduce the number of dimensions representing each cell. The number of components from this analysis used for the elbow of a scree plot. Based on differential expression between neighboring clusters in the samples and biological relevance, the numbers of clusters were selected. The different clusters in a sample were visualized using t-distributed stochastic neighbor embedding of the principal components as implemented in Seurat. The specific cell-type identities for each cluster were determined manually using a compiled panel of available known immune cells and other cell specific marker expression.

### Additional datasets

Obese adipose tissue single-cell data [23] (GSE117176 from GSM3272967 obese ATM) was reanalyzed similarly as mentioned above to infer additional cell type information for the genes in the tissue-specific network genes.

### Cell type assignments to network genes from single-cell data

Adipose tissue genes from the transkingdom network were classified into specific cell types based on primarily by significant fold change in a specific cell cluster and additionally by individual genes ranked average expression in a specific cell cluster. Rules for which cell a gene belongs to is determined by clusters of given cells over all other cells with *p*-value (<0.05) and fold change (log2FC > 0.25). This was done for all genes in the adipose tissue network. It was followed as a basic rule to assign a gene to a cell type. Alternatively, a higher expression in the cell cluster (and an optional fold change (log2FC > 0.25, *p*-value < 0.05) for a gene was assigned with that specific cell type. Here ranking by average expression for each gene in the clusters helps to determine its cluster specificity by higher expression in that cell type than another. This was implemented for evaluating highest cluster average expression of a gene, among all other cell clusters in network. A similar method was followed for the liver tissue network as well as to assign the cell type information to individual genes.

### Microbiota-dependent gene expression in the adipose tissue

Methods were followed as described previously in the first part of this paper [22].

### Defining mitochondrial genes in the adipose tissue

Methods were followed as detailed previously [22]. Gene Ontology (GO) analysis was conducted using Metascape (https://metascape.org/gp/index.html#/main/step1) [63]. Adipose tissue genes in the network with TXN treatment (effect *p*-value <0.05) were analyzed with GO biological process against mouse databases.

### Statistical analysis

Both transcriptome and microbiome data were relativized per million, quantile normalized, and log2-transformed prior to analysis. To avoid log2 values of 0, a pseudocount of 1 was added to each normalized CPM before log2 transformation. Bile acids and phenotypic data were median normalized across all samples. As the data did not follow a normal distribution, R was used to perform Mann–Whitney comparisons between the groups (HFD vs LFD, XN vs HFD, and TXN vs HFD). Two-tailed tests were performed unless otherwise noted. Correlations were performed as described in the previous section, “Transkingdom multi-organ network construction and interrogation”. Graphpad v9.4.1 was used to perform chi-square tests. Analysis of the comparison of the observed *Oscillibacter* sp.’s BiBC compared to the distribution of BiBCs found in the random networks was performed using a one-sample Wilcoxon test with the function wilcox.test() in the R stats package v3.6.2. Additional details of statistical analyses are described in the corresponding figure legends.

### Supplementary Information

**Additional file 1: Supplementary Figure S1.** Myeloid cells are the primary cell type affected by TXN in the adipose tissue. **Supplementary Figure S2.** TXN treatment alters fecal microbiota composition and some *Oscillibacter* features. **Supplementary Figure S3.**  TXN treatment increases mitochondrial gene expression. **Figure S4.** Identification of overlapping gene expression between adipose tissue from TXN-treated mice vs. *O.valericigenes* supernatant-treated macrophage cell lines. **Figure S5.** Gene expression from in vitro titration of *O. valericigenes* and *L. gasseri* cell free supernatant on a IMM cell line.

**Additional file 2: Table S1.** Number of parameters per data type in each category in the network. **Table S2.** Summary table of parameters, including statistical information, cell type information, microbiota dependence/independence for genes, abundance of microbes, and network information. **Table S3.** IMM/RAW cell with *Oscillibacter* supplementation (data from GSE203488, GSE203516). **Table S4.** In vivo validation of *O. valericigenes* gavage (data from GSE215226). **Table S5.** RT-PCR  data from *in vitro* cell free  *Oscillibacter* supernatant titration on IMM cells.

**Additional file 3.** Processed data of the single cell RNA sequencing from adipose tissue (WAT_filtered_feature_bc_matrix.h5). 

## Data Availability

All source data and materials are provided with this paper. Raw and processed data has submitted to Gene Expression Omnibus (GEO) [64]. The sequencing of epidydimal adipose tissue in mice fed a normal diet with/without *O. valericigenes* gavage can be found under the GEO accession number GSE215226. The adipose tissue single-cell data is available as a supplementary file. RNA-Seq data of IMM cells treated with Oscillibacter supernatant: GSE203488. RNA-Seq data of RAW 264.7 cells treated with Oscillibacter supernatant: GSE203516. RNA-Seq data of liver of mice: GSE164636. RNA-Seq data of ileum and epidydimal adipose tissue of mice: GSE219235. Microbiome 16S rRNA DNA sequencing: Sequence Read Archive SUB10676151.

## References

[1] Belete R, Ataro Z, Abdu A, Sheleme M (2021). Global prevalence of metabolic syndrome among patients with type I diabetes mellitus: a systematic review and meta-analysis. Diabetol Metab Syndr.

[2] Eslam M, Sanyal AJ, George J, International Consensus P (2020). MAFLD: a consensus-driven proposed nomenclature for metabolic associated fatty liver disease. Gastroenterology.

[3] Fouad Y, Elwakil R, Elsahhar M, Said E, Bazeed S, Ali Gomaa A, Hashim A, Kamal E, Mehrez M, Attia D (2021). The NAFLD-MAFLD debate: eminence vs evidence. Liver Int.

[4] Nucera S, Ruga S, Cardamone A, Coppoletta AR, Guarnieri L, Zito MC, Bosco F, Macrì R, Scarano F, Scicchitano M (2022). MAFLD progression contributes to altered thalamus metabolism and brain structure. Sci Rep.

[5] Pérez-Muñoz EP, Antunes-Ricardo M, Martínez-Ávila M, Guajardo-Flores D (2022). Eryngium species as a potential ally for treating metabolic syndrome and diabetes. Front Nutr.

[6] Chiva-Blanch G, Badimon L (2017). Effects of polyphenol intake on metabolic syndrome: current evidences from human trials. Oxid Med Cell Longev.

[7] Liu K, Luo M, Wei S (2019). The bioprotective effects of polyphenols on metabolic syndrome against oxidative stress: evidences and perspectives. Oxid Med Cell Longev.

[8] Rajha HN, Paule A, Aragonès G, Barbosa M, Caddeo C, Debs E, Dinkova R, Eckert GP, Fontana A, Gebrayel P (2022). Recent advances in research on polyphenols: effects on microbiota, metabolism, and health. Mol Nutr Food Res.

[9] Lee I-S, Lim J, Gal J, Kang JC, Kim HJ, Kang BY, Choi HJ (2011). Anti-inflammatory activity of xanthohumol involves heme oxygenase-1 induction via NRF2-ARE signaling in microglial BV2 cells. Neurochem Int.

[10] Mills DM, Samuels J, Hawkins M, Gavre A, Shashidharamurthy R, Rayalam S (2019). Anti-inflammatory effects of xanthohumol in RAW 264.7 macrophages are mediated through the activation of AMP kinase. J Immunol.

[11] Vazquez-Cervantes GI, Ortega DR, Blanco Ayala T, Pérez de la Cruz V, Esquivel DFG, Salazar A, Pineda B: Redox and anti-inflammatory properties from hop components in beer-related to neuroprotection. Nutrients 2021, 13(6). 10.3390/nu13062000 PMC8226943 34200665

[12] Costa R, Rodrigues I, Guardao L, Rocha-Rodrigues S, Silva C, Magalhaes J, Ferreira-de-Almeida M, Negrao R, Soares R (2017). Xanthohumol and 8-prenylnaringenin ameliorate diabetic-related metabolic dysfunctions in mice. J Nutr Biochem.

[13] Miranda CL, Johnson LA, de Montgolfier O, Elias VD, Ullrich LS, Hay JJ, Paraiso IL, Choi J, Reed RL, Revel JS (2018). Non-estrogenic xanthohumol derivatives mitigate insulin resistance and cognitive impairment in high-fat diet-induced obese mice. Sci Rep.

[14] Miyata S, Inoue J, Shimizu M, Sato R (2015). Xanthohumol improves diet-induced obesity and fatty liver by suppressing sterol regulatory element-binding protein (SREBP) activation. J Biol Chem.

[15] Takahashi K, Osada K (2017). Effect of dietary purified xanthohumol from Hop (Humulus lupulus L.) pomace on adipose tissue mass, fasting blood glucose level, and lipid metabolism in KK-Ay mice. J Oleo Sci.

[16] Zhang Y, Bobe G, Miranda CL, Lowry MB, Hsu VL, Lohr CV, Wong CP, Jump DB, Robinson MM, Sharpton TJ, et al. Tetrahydroxanthohumol, a xanthohumol derivative, attenuates high-fat diet-induced hepatic steatosis by antagonizing PPARγ. Elife. 2021;10:e66398. 10.7554/eLife.66398 PMC8205491 34128467

[17] Zhang Y, Bobe G, Revel JS, Rodrigues RR, Sharpton TJ, Fantacone ML, Raslan K, Miranda CL, Lowry MB, Blakemore PR (2020). Improvements in metabolic syndrome by xanthohumol derivatives are linked to altered gut microbiota and bile acid metabolism. Mol Nutr Food Res.

[18] Logan IE, Shulzhenko N, Sharpton TJ, Bobe G, Liu K, Nuss S, Jones ML, Miranda CL, Vasquez-Perez S, Pennington JM (2021). Xanthohumol requires the intestinal microbiota to improve glucose metabolism in diet-induced obese mice. Mol Nutr Food Res.

[19] Dong X, Yambartsev A, Ramsey SA, Thomas LD, Shulzhenko N, Morgun A (2015). Reverse enGENEering of regulatory networks from big data: a roadmap for biologists. Bioinform Biol Insights.

[20] Rodrigues RR, Gurung M, Li Z, García-Jaramillo M, Greer R, Gaulke C, Bauchinger F, You H, Pederson JW, Vasquez-Perez S (2021). Transkingdom interactions between Lactobacilli and hepatic mitochondria attenuate western diet-induced diabetes. Nat Commun.

[21] Lam KC, Vyshenska D, Hu J, Rodrigues RR, Nilsen A, Zielke RA, Brown NS, Aarnes E-K, Sikora AE, Shulzhenko N (2018). Transkingdom network reveals bacterial players associated with cervical cancer gene expression program. PeerJ.

[22] Li Z, Gurung M, Rodrigues RR, Padiadpu J, Newman NK, Manes NP, Pederson JW, Greer RL, Vasquez-Perez S, You H, et al. Microbiota and adipocyte mitochondrial damage in type 2 diabetes are linked by Mmp12+ macrophages. J Exp Med. 2022;219(7):e20220017. 10.1084/jem.20220017 PMC9170383 35657352

[23] Li C, Menoret A, Farragher C, Ouyang Z, Bonin C, Holvoet P, Vella AT, Zhou B. Single cell transcriptomics based-MacSpectrum reveals novel macrophage activation signatures in diseases. JCI Insight. 2019;5(10):e126453. 10.1172/jci.insight.126453 PMC6542613 30990466

[24] Morgun A, Dzutsev A, Dong X, Greer RL, Sexton DJ, Ravel J, Schuster M, Hsiao W, Matzinger P, Shulzhenko N. Uncovering effects of antibiotics on the host and microbiota using transkingdom gene networks. Gut. 2015;64:1732–43. 10.1136/gutjnl-2014-308820 PMC5166700 25614621

[25] Mao G, Zhao Y, Kang X, Li Z, Zhang Y, Wang X, Sun F, Sankaran K, Zhang XC (2016). Crystal structure of E. coli lipoprotein diacylglyceryl transferase. Nat Commun..

[26] Rath S, Sharma R, Gupta R, Ast T, Chan C, Durham TJ, Goodman RP, Grabarek Z, Haas ME, Hung WH (2021). MitoCarta3. 0: an updated mitochondrial proteome now with sub-organelle localization and pathway annotations. Nucleic Acids Res.

[27] Scalcon V, Bindoli A, Rigobello MP (2018). Significance of the mitochondrial thioredoxin reductase in cancer cells: an update on role, targets and inhibitors. Free Radic Biol Med.

[28] Raza H, Robin M-A, Fang J-K, Avadhani NG (2002). Multiple isoforms of mitochondrial glutathione S-transferases and their differential induction under oxidative stress. Biochem J.

[29] Shi L, Tu BP (2015). Acetyl-CoA and the regulation of metabolism: mechanisms and consequences. Curr Opin Cell Biol.

[30] Mahli A, Seitz T, Freese K, Frank J, Weiskirchen R, Abdel-Tawab M, Behnam D, Hellerbrand C. Therapeutic application of micellar solubilized xanthohumol in a western-type diet-induced mouse model of obesity, diabetes and non-alcoholic fatty liver disease. Cells. 2019;8(4):359. 10.3390/cells8040359 PMC6523748 30999670

[31] Miranda CL, Elias VD, Hay JJ, Choi J, Reed RL, Stevens JF (2016). Xanthohumol improves dysfunctional glucose and lipid metabolism in diet-induced obese C57BL/6J mice. Arch Biochem Biophys.

[32] Nozawa H (2005). Xanthohumol, the chalcone from beer hops (Humulus lupulus L.), is the ligand for farnesoid X receptor and ameliorates lipid and glucose metabolism in KK-A(y) mice. Biochem Biophys Res Commun.

[33] Karimi-Sales E, Mohaddes G, Alipour MR (2018). Chalcones as putative hepatoprotective agents: preclinical evidence and molecular mechanisms. Pharmacol Res.

[34] Weiskirchen R, Mahli A, Weiskirchen S, Hellerbrand C (2015). The hop constituent xanthohumol exhibits hepatoprotective effects and inhibits the activation of hepatic stellate cells at different levels. Front Physiol.

[35] Kirkwood JS, Legette LL, Miranda CL, Jiang Y, Stevens JF (2013). A metabolomics-driven elucidation of the anti-obesity mechanisms of xanthohumol. J Biol Chem.

[36] Paraiso IL, Mattio LM, Alcázar Magaña A, Choi J, Plagmann LS, Redick MA, Miranda CL, Maier CS, Dallavalle S, Kioussi C (2021). Xanthohumol pyrazole derivative improves diet-induced obesity and induces energy expenditure in high-fat diet-fed mice. ACS Pharmacol Transl Sci.

[37] Yui K, Kiyofuji A, Osada K (2014). Effects of xanthohumol-rich extract from the hop on fatty acid metabolism in rats fed a high-fat diet. J Oleo Sci.

[38] Doddapattar P, Radović B, Patankar JV, Obrowsky S, Jandl K, Nusshold C, Kolb D, Vujić N, Doshi L, Chandak PG (2013). Xanthohumol ameliorates atherosclerotic plaque formation, hypercholesterolemia, and hepatic steatosis in ApoE-deficient mice. Mol Nutr Food Res.

[39] Vetrani C, Di Nisio A, Paschou SA, Barrea L, Muscogiuri G, Graziadio C, Savastano S, Colao A, On Behalf Of The Obesity Programs Of Nutrition Education R, Assessment Opera G. From gut microbiota through low-grade inflammation to obesity: key players and potential targets. Nutrients. 2022;14(10):2103. 10.3390/nu14102103 PMC9145366 35631244

[40] Shin H-S, Xu F, Bagchi A, Herrup E, Prakash A, Valentine C, Kulkarni H, Wilhelmsen K, Warren S, Hellman J (2011). Bacterial lipoprotein TLR2 agonists broadly modulate endothelial function and coagulation pathways in vitro and in vivo. J Immunol.

[41] Song WS, Jeon YJ, Namgung B, Hong M, Yoon S-I (2017). A conserved TLR5 binding and activation hot spot on flagellin. Sci Rep.

[42] Hill DA, Lim H-W, Kim YH, Ho WY, Foong YH, Nelson VL, Nguyen HCB, Chegireddy K, Kim J, Habertheuer A (2018). Distinct macrophage populations direct inflammatory versus physiological changes in adipose tissue. Proc Natl Acad Sci U S A.

[43] Jaitin DA, Adlung L, Thaiss CA, Weiner A, Li B, Descamps H, Lundgren P, Bleriot C, Liu Z, Deczkowska A (2019). Lipid-Associated macrophages control metabolic homeostasis in a Trem2-dependent manner. Cell.

[44] Harasymowicz NS, Rashidi N, Savadipour A, Wu C-L, Tang R, Bramley J, Buchser W, Guilak F (2021). Single-cell RNA sequencing reveals the induction of novel myeloid and myeloid-associated cell populations in visceral fat with long-term obesity. FASEB J.

[45] Silva HM, Báfica A, Rodrigues-Luiz GF, Chi J, Santos PDeA, Reis BS, Hoytema van Konijnenburg DP, Crane A, Arifa RDN, Martin P (2019). Vasculature-associated fat macrophages readily adapt to inflammatory and metabolic challenges. J Exp Med.

[46] Kratz M, Coats BR, Hisert KB, Hagman D, Mutskov V, Peris E, Schoenfelt KQ, Kuzma JN, Larson I, Billing PS (2014). Metabolic dysfunction drives a mechanistically distinct proinflammatory phenotype in adipose tissue macrophages. Cell Metab.

[47] Agus A, Clément K, Sokol H (2021). Gut microbiota-derived metabolites as central regulators in metabolic disorders. Gut.

[48] Dabke K, Hendrick G, Devkota S (2019). The gut microbiome and metabolic syndrome. J Clin Investig.

[49] Gurung M, Li Z, You H, Rodrigues R, Jump DB, Morgun A, Shulzhenko N (2020). Role of gut microbiota in type 2 diabetes pathophysiology. EBioMedicine.

[50] Jung M-J, Lee J, Shin N-R, Kim M-S, Hyun D-W, Yun J-H, Kim PS, Whon TW, Bae J-W (2016). Chronic repression of mTOR complex 2 induces changes in the gut microbiota of diet-induced obese mice. Sci Rep.

[51] Fiebiger U, Bereswill S, Heimesaat MM (2016). Dissecting the interplay between intestinal microbiota and host immunity in health and disease: lessons learned from germfree and gnotobiotic animal models. Eur J Microbiol Immunol.

[52] Round JL, Mazmanian SK (2009). The gut microbiota shapes intestinal immune responses during health and disease. Nat Rev Immunol.

[53] Tran HQ, Bretin A, Adeshirlarijaney A, San Yeoh B, Vijay-Kumar M, Zou J, Denning TL, Chassaing B, Gewirtz AT (2020). “Western diet”-induced adipose inflammation requires a complex gut microbiota. Cell Mol Gastroenterol Hepatol.

[54] Paraiso IL, Tran TQ, Magana AA, Kundu P, Choi J, Maier CS, Bobe G, Raber J, Kioussi C, Stevens JF (2021). Xanthohumol ameliorates diet-induced liver dysfunction via farnesoid X receptor-dependent and independent signaling. Front Pharmacol.

[55] Callahan BJ, McMurdie PJ, Rosen MJ, Han AW, Johnson AJA, Holmes SP (2016). DADA2: high-resolution sample inference from Illumina amplicon data. Nat Methods.

[56] Cole JR, Wang Q, Fish JA, Chai B, McGarrell DM, Sun Y, Brown CT, Porras-Alfaro A, Kuske CR, Tiedje JM (2014). Ribosomal Database Project: data and tools for high throughput rRNA analysis. Nucleic Acids Res.

[57] De Nardo D, Labzin LI, Kono H, Seki R, Schmidt SV, Beyer M, Xu D, Zimmer S, Lahrmann C, Schildberg FA (2014). High-density lipoprotein mediates anti-inflammatory reprogramming of macrophages via the transcriptional regulator ATF3. Nat Immunol.

[58] Balduzzi S, Rücker G, Schwarzer G (2019). How to perform a meta-analysis with R: a practical tutorial. Evid-Based Mental Health.

[59] Yambartsev A, Perlin MA, Kovchegov Y, Shulzhenko N, Mine KL, Dong X, Morgun A (2016). Unexpected links reflect the noise in networks. Biol Direct.

[60] Chunikhina E, Logan P, Kovchegov Y, Yambartsev A, Mondal D, Morgun A (2022). The C-SHIFT algorithm for normalizing covariances. IEEE/ACM Transactions on Computational Biology and Bioinformatics.

[61] Kahalehili HM, Newman NK, Pennington JM, Kolluri SK, Kerkvliet NI, Shulzhenko N, Morgun A, Ehrlich AK (2020). Dietary indole-3-carbinol activates AhR in the Gut, Alters Th17-microbe interactions, and exacerbates insulitis in NOD mice. Front Immunol.

[62] Hao Y, Hao S, Andersen-Nissen E, Mauck WM, Zheng S, Butler A, Lee MJ, Wilk AJ, Darby C, Zager M (2021). Integrated analysis of multimodal single-cell data. Cell.

[63] Zhou Y, Zhou B, Pache L, Chang M, Khodabakhshi AH, Tanaseichuk O, Benner C, Chanda SK (2019). Metascape provides a biologist-oriented resource for the analysis of systems-level datasets. Nat Commun.

[64] Barrett T, Wilhite SE, Ledoux P, Evangelista C, Kim IF, Tomashevsky M, Marshall KA, Phillippy KH, Sherman PM, Holko M (2012). NCBI GEO: archive for functional genomics data sets—update. Nucleic Acids Res.

