# Supplementary material for: Reducing gut microbiome-driven adipose tissue inflammation alleviates metabolic syndrome
Source: Microbiome. 2023 Sep 21;11:208. doi: 10.1186/s40168-023-01637-4 (PMC10512512; doi:10.1186/s40168-023-01637-4)
Supplement: Supplementary file 2 — Additional file 1: Supplementary Figure S1. Myeloid cells are the primary cell type affected by TXN in the adipose tissue. Supplementary Figure S2. TXN treatment alters fecal microbiota composition and some Oscillibacter features. Supplementary Figure S3. TXN treatment increases mitochondrial gene expression. Figure S4. Identification of overlapping gene expression between adipose tissue from TXN-treated mice vs. O.valericigenes supernatant-treated macrophage cell lines. Figure S5. Gene expression from in vitro titration of O. valericigenes and L. gasseri cell free supernatant on a IMM cell line. [file 40168_2023_1637_MOESM1_ESM.pdf]

A

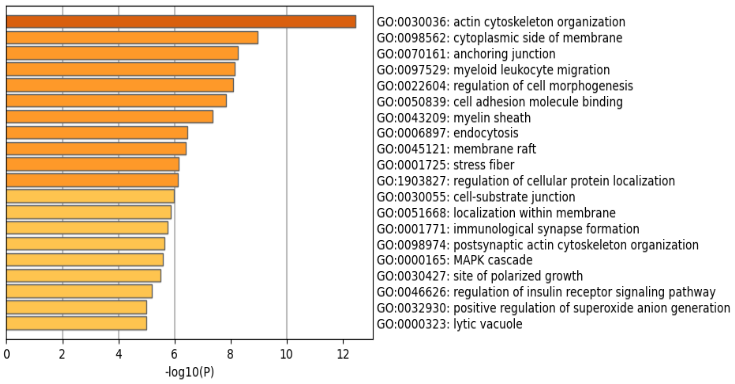

B

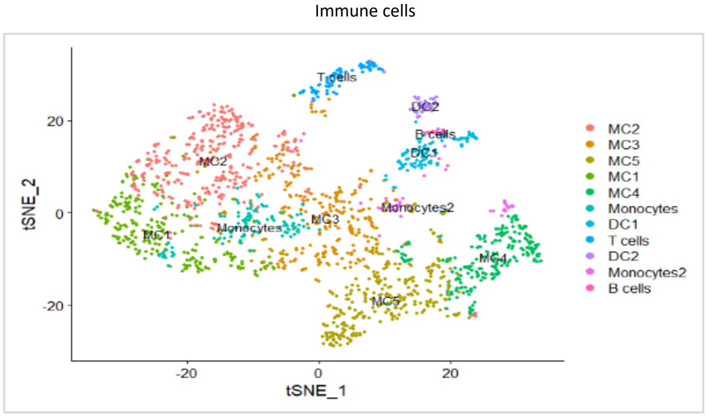

Adipose tissue CD45 + cells, GSE117176-GSM3272967 (obese ATM)

C

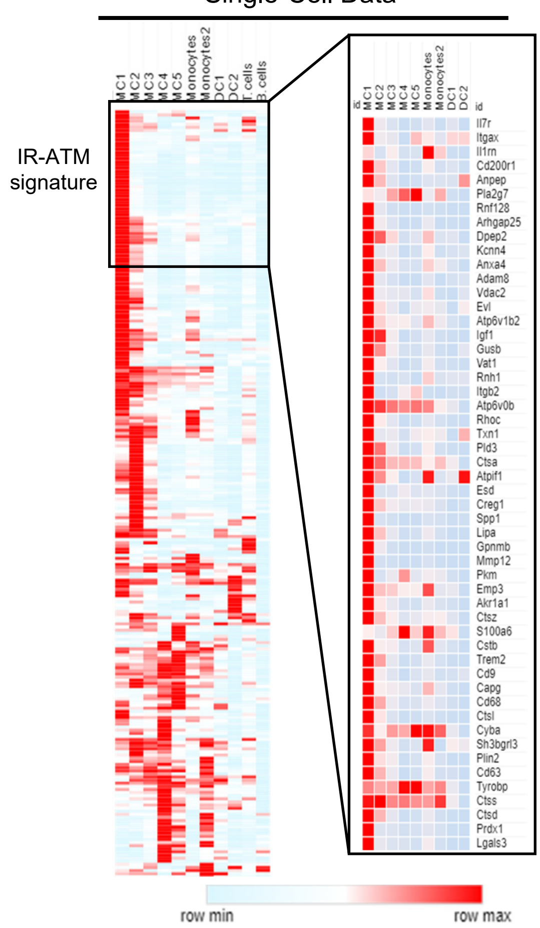

D

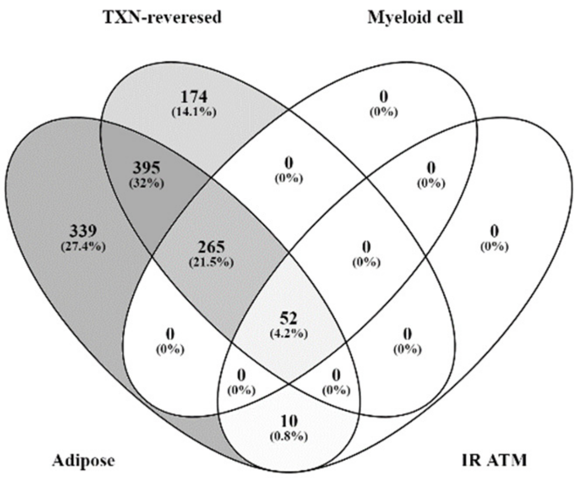

Supplementary Figure S1 – Myeloid cells are the primary cell type affected by TXN in the adipose tissue.

A. Functional enrichment of genes belonging to myeloid cells and improved by TXN treatment indicates significant enrichment of myeloid cell activation, endocytosis and insulin receptor pathways.

B. T-SNE plot shows re-analyzed clusters of different cell types from a previously published dataset (GSE117176) containing single cell sequencing of murine adipose tissue stromal cells on a high fat diet. The immune cell clusters are labeled based on the specific immune cell markers derived from the reanalysis.

C. Average expression from the validation single cell study (GSE117176) for each of the genes assigned to a specific cell type is shown. Many of the adipose tissue genes in our whole tissue RNA-seq study were assigned to myeloid cells, primarily the metabolic macrophage (IR-ATM) type, named here ‘MC1’ cells. The zoomed in heatmap in the right panel shows the higher average expression of IR-ATM signature genes in the MC1 group.

D. The Venn diagram represents the adipose tissue genes from the network and shows the genes reversed by TXN that were assigned to myeloid cells and/or IR-ATMs. The IR-ATM gene signature is significantly reversed by TXN (52/62) as shown.

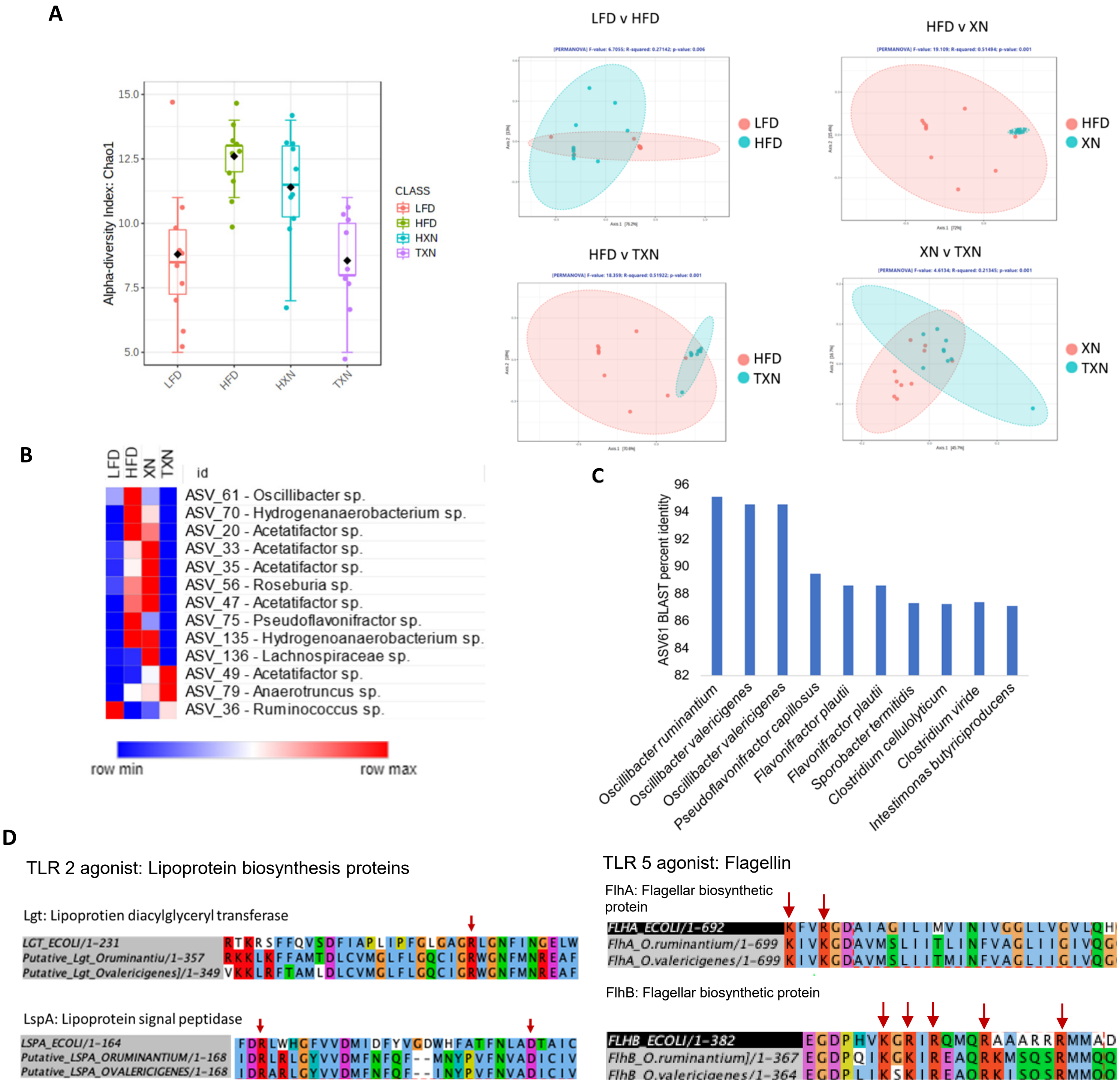

Supplementary Figure S2 – TXN treatment alters fecal microbiota composition and some *Oscillibacter* features.

A. Left panel: Comparison of alpha diversity across treatment groups. Right panel: Beta diversity (pairwise PERMANOVA between treatment groups).

B. Abundance of ASVs for each treatment group. ASVs plotted are from those in the top BiBC in Figure 3E. The colors represent the mean quantile normalized counts per million of each group, relative to the other groups in the row (e.g., the darkest blue color indicates the lowest mean while dark red indicates the highest).

C. BLAST percent identity comparison of ASV61 indicates high similarity to both *O. ruminantium* and *O. valericigenes* as the top three hits. Subsequent hits show a much-reduced similarity. Notably, none of the subsequent hits are from the *Oscillibacter* genus.

D. ClustalO multiple sequence alignment shows TLR2/TLR5 agonist protein sequences of *Oscillibacter* aligned with respective *E. coli* sequences containing conserved domains, which also have same functional residues. The red arrows indicate the functional residues in the protein sequence alignment.

A

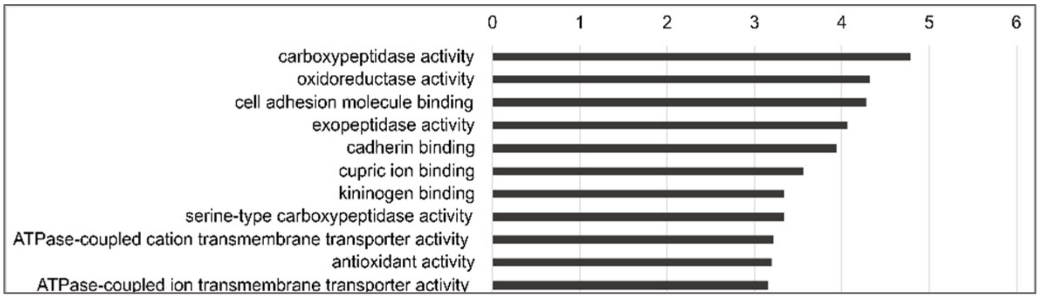

B

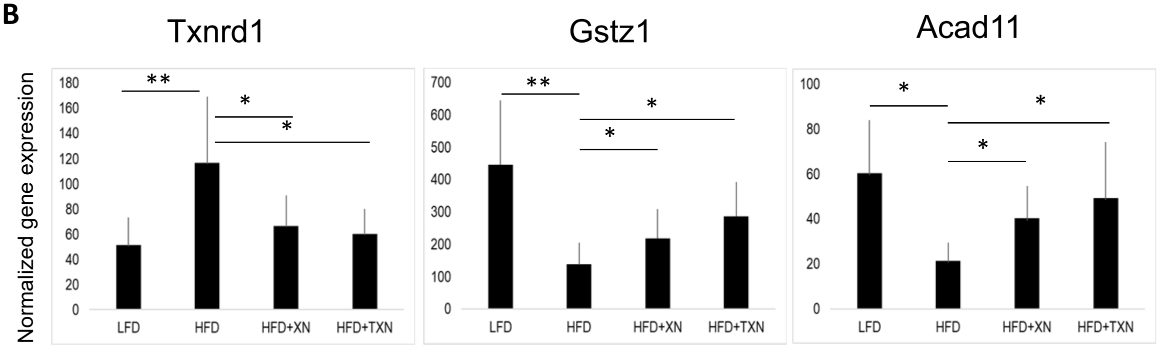

Supplementary Figure S3 – TXN treatment increases mitochondrial gene expression

A. The top BiBC mitochondrial genes in the adipose tissue network are enriched for oxidoreductase activity and antioxidant activity. Values shown are in normalized counts per million.

B. Expression levels for representative mitochondrial genes significantly reversed by TXN that comprise top BiBC in the adipose tissue network.

A

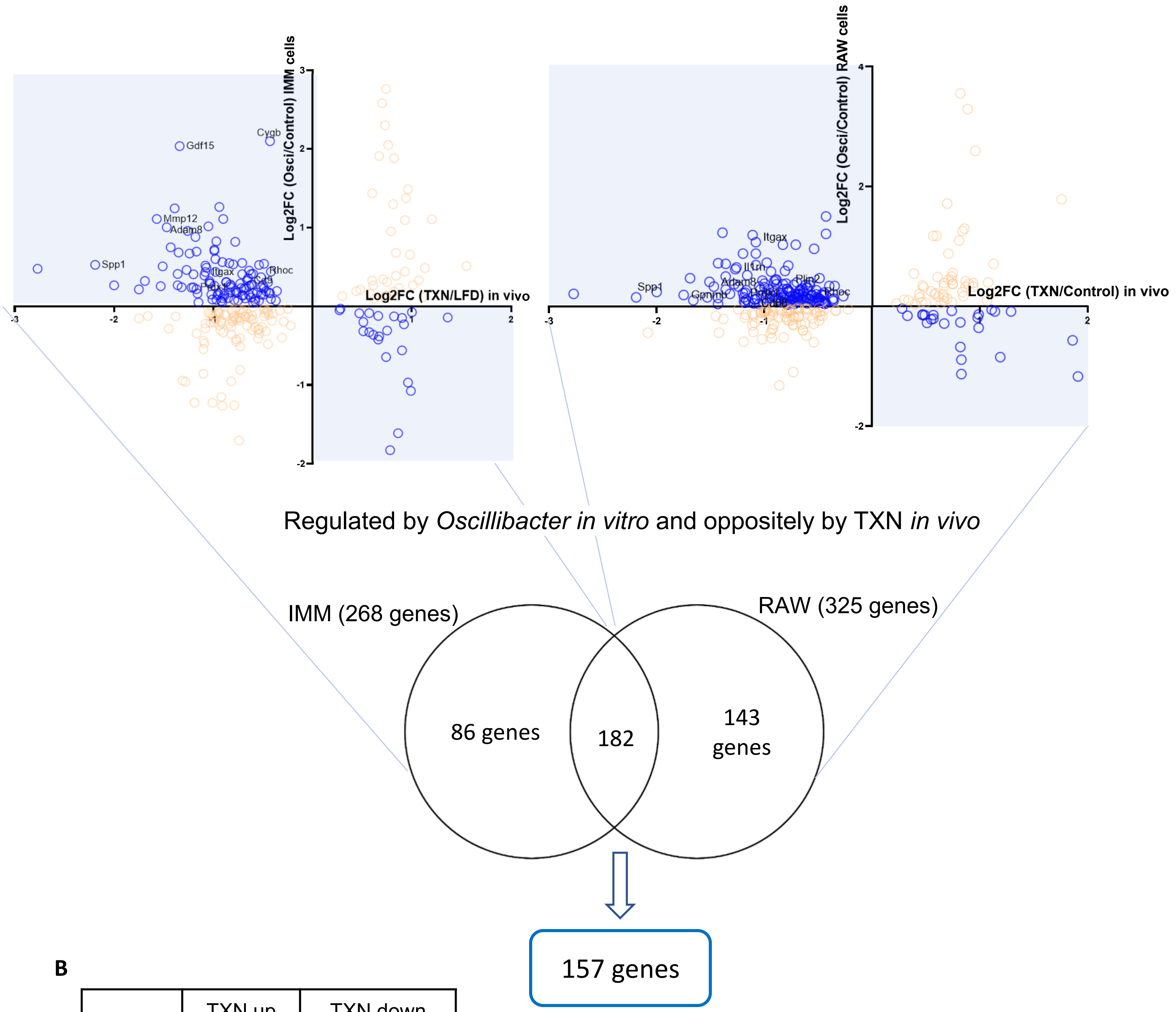

B

	TXN up	TXN down
Osc up	21	**79**
Osc down	**6**	51

Fisher P-value 0.047

Figure S4 – Identification of overlapping gene expression between adipose tissue from TXN-treated mice vs. O. valericigenes supernatant-treated macrophage cell lines.

A. Finding the overlap of the genes differentially expressed in O. valericigenes treated IMM cells (graph on top left) and O. valericigenes treated RAW 364.7 cells (graph on top right) that were differentially expressed in the opposite direction with TXN treatment in vivo (blue shaded area). Circles are genes assigned to the TXN-reversed category in vivo. Mann-Whitney P < 0.1, Oscillibacter treated vs. untreated cells. Meta-analysis of the IMM and RAW 264.7 cells yields 157 significant genes (Fisher's combined P < 0.05).

B. Fisher analysis results for Figure S4A.

Figure S5

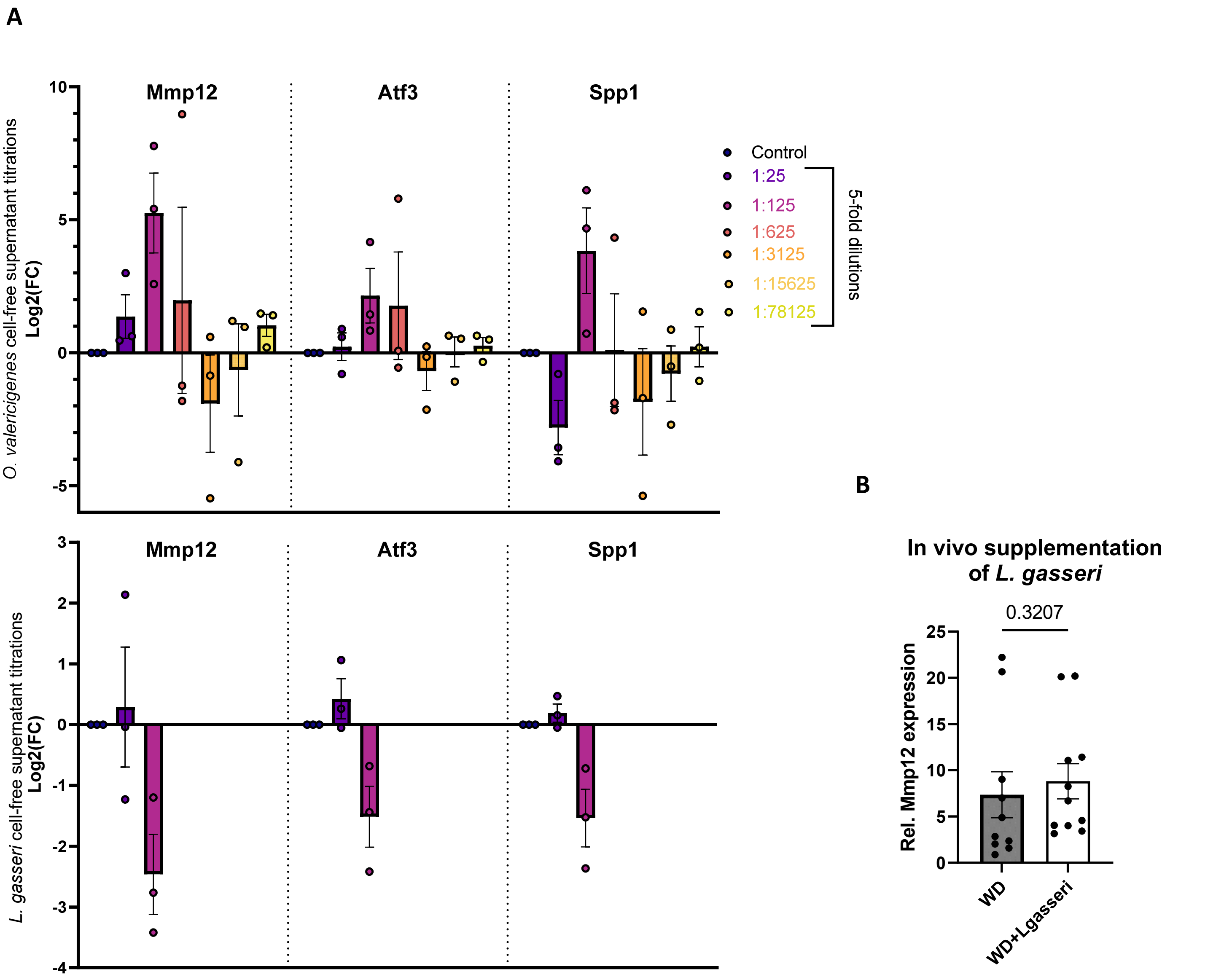

Figure S5 - Gene expression from in vitro **titration of *O. valericigenes* and *L. gasseri* cell free supernatant on a IMM cell line.**

A. Gene expression data by qPCR are median normalized, log2 transformed fold changes (treatment/control group). Bars are group means, and error bars are SEM. Individual dots correspond to individual experiments (n=3).

B. Relative expression of Mmp12 in adipose tissue of mice supplemented with *L. gasseri.* The data is from two independent experiments, dots representing individual mice (One-sided, unpaired t-test).
